# A comparison of approximation techniques for variance-based sensitivity analysis of biochemical reaction systems

**DOI:** 10.1186/1471-2105-11-246

**Published:** 2010-05-12

**Authors:** Hong-Xuan Zhang, John Goutsias

**Affiliations:** 1 Whitaker Biomedical Engineering Institute, The Johns Hopkins University, Baltimore, MD 21218, USA

## Abstract

**Background:**

Sensitivity analysis is an indispensable tool for the analysis of complex systems. In a recent paper, we have introduced a thermodynamically consistent variance-based sensitivity analysis approach for studying the robustness and fragility properties of biochemical reaction systems under uncertainty in the standard chemical potentials of the activated complexes of the reactions and the standard chemical potentials of the molecular species. In that approach, key sensitivity indices were estimated by Monte Carlo sampling, which is computationally very demanding and impractical for large biochemical reaction systems. Computationally efficient algorithms are needed to make variance-based sensitivity analysis applicable to realistic cellular networks, modeled by biochemical reaction systems that consist of a large number of reactions and molecular species.

**Results:**

We present four techniques, derivative approximation (DA), polynomial approximation (PA), Gauss-Hermite integration (GHI), and orthonormal Hermite approximation (OHA), for *analytically *approximating the variance-based sensitivity indices associated with a biochemical reaction system. By using a well-known model of the mitogen-activated protein kinase signaling cascade as a case study, we numerically compare the approximation quality of these techniques against traditional Monte Carlo sampling. Our results indicate that, although DA is computationally the most attractive technique, special care should be exercised when using it for sensitivity analysis, since it may only be accurate at low levels of uncertainty. On the other hand, PA, GHI, and OHA are computationally more demanding than DA but can work well at high levels of uncertainty. GHI results in a slightly better accuracy than PA, but it is more difficult to implement. OHA produces the most accurate approximation results and can be implemented in a straightforward manner. It turns out that the computational cost of the four approximation techniques considered in this paper is orders of magnitude smaller than traditional Monte Carlo estimation. Software, coded in MATLAB^®^, which implements all sensitivity analysis techniques discussed in this paper, is available free of charge.

**Conclusions:**

Estimating variance-based sensitivity indices of a large biochemical reaction system is a computationally challenging task that can only be addressed via approximations. Among the methods presented in this paper, a technique based on orthonormal Hermite polynomials seems to be an acceptable candidate for the job, producing very good approximation results for a wide range of uncertainty levels in a fraction of the time required by traditional Monte Carlo sampling.

## Background

Sensitivity analysis is an indispensable tool for the analysis of complex systems [1,2]. It is routinely used to investigate how uncertainty in input variables affects uncertainty in system response and to quantify the relative importance of the input variables in influencing the response. In addition to many other areas of science and engineering, sensitivity analysis is used in systems biology to investigate the robustness and fragility properties of cellular systems, such as signaling, gene regulation, and metabolic networks [3-11], as well as in systems pharmacology [12], for designing novel pharmacological intervention strategies and for understanding drug action [13,14].

To study the sensitivity properties of a biochemical reaction system, such as a signaling network, we must construct a mathematical model that relates uncertainty in key biochemical factors of interest to a biologically relevant system response, and develop techniques for determining how factor uncertainty affects the system response. Since biochemical reaction systems are subject to physical laws, an important requirement is that sensitivity analysis must satisfy important thermodynamic constraints, such as the principle of detailed balance [15]. Bearing these in mind, we have proposed in [16] a probabilistic sensitivity analysis approach for biochemical reaction systems that uses the standard chemical potentials of the activated complexes of the underlying reactions and molecular species as the biochemical factors of interest and propagates factor uncertainty to a given system response in a thermodynamically consistent manner. Moreover, we have adopted a formal statistical approach to sensitivity analysis, known as variance-based sensitivity analysis [2,17-19], which uses a set of indices to quantify the contribution of individual biochemical factors to the variance of the system response.

Unfortunately, it is not in general possible to analytically evaluate variance-based sensitivity indices. As a consequence, these indices are estimated by Monte Carlo sampling [2,16,18,20], which requires evaluation of the system response at each sample. A major drawback of this approach is its slow rate of convergence. As a matter of fact, the error produced by a naive Monte Carlo estimation approach decreases with an error rate of *O*(1/), where *L *is the number of Monte Carlo samples used [21]. Hence, accurate estimation of the sensitivity indices requires a large number of Monte Carlo samples and, therefore, a large number of system response evaluations. This makes Monte Carlo estimation of variance-based sensitivity indices computationally very expensive, especially in the case of biochemical reaction systems comprised of a large number of reactions and molecular species.

To reduce the computational burden of Monte Carlo estimation, it is imperative that we develop techniques which can produce sufficiently accurate estimates of the sensitivity indices in a fraction of the time required by Monte Carlo sampling. In this paper, we present four such techniques and apply them to a well-known biochemical reaction model of the mitogen-activated protein kinase (MAPK) signalling cascade. The first technique is based on a second-order Taylor series expansion of the response function and is an extension of the first-order derivative-based approach for variance-based sensitivity analysis discussed in [2,18,19,22] by including second-order derivative terms. The other approximation techniques are based on the high-dimensional model representation (HDMR) schemes developed by H. Rabitz and his coworkers [23-25]. We use analytical derivations, provided in the Additional file 1 accompanying this paper, and sensitivity analysis results generated by the four methods, to clarify the relative merits of each approximation technique and produce useful insights on when these techniques can be used for sensitivity analysis of biochemical reaction systems. We have coded the sensitivity analysis techniques discussed in this paper using MATLAB^®^. Interested readers can request a copy of the software, and the entire set of data obtained with this software, by contacting the corresponding author.

We should mention here that, in systems biology, the most commonly used sensitivity analysis techniques are based on derivatives of molecular concentrations or other system responses, known as control coefficients [3]. These differential methods are based on a Taylor series approximation of the response function and, as such, are subject to several drawbacks that must be carefully considered before applying them to problems of systems biology. For example, derivative-based sensitivity indices assess the sensitivity properties of a biochemical reaction system around a set of reference input values. Their performance usually depends on the particular choice of these values, due to the nonlinear nature of the response function. For the results to be relevant, the reference values must be the true values, which are usually not known in practice. As a consequence, derivative-based sensitivity analysis techniques are limited by the quality of the underlying Taylor series approximation. Moreover, and due to our difficulty in accurately evaluating high-order derivatives, differential sensitivity analysis techniques are usually limited in practice to assessing the effect of one input factor on the system response, by keeping all other factors fixed to their reference values. This is usually not adequate, since we are most often interested in the effects of multiple biochemical factors on the system response. Finally, traditional differential analysis cannot cope with probabilistic uncertainty in biochemical factor values, unless it is combined with variance-based sensitivity analysis (as it is done by the first approximation technique considered in this paper). It turns out that variance-based sensitivity analysis does not depend on the additivity or linearity of the system model and can be naturally used to quantify the simultaneous effect of probabilistic biochemical factor uncertainty on the system response [2,18]. For this reason, it provides a very attractive and powerful approach for sensitivity analysis of biochemical reaction systems.

We should finally mention that a number of alternative approximation techniques for variance-based sensitivity analysis have been proposed in the literature [26-29]. In these techniques, the original response function is approximated by a surrogate function and the sensitivity indices are then estimated by Monte Carlo sampling based on that function. Reduction in computations is achieved by the fact that the time required for computing the system response at each Monte Carlo iteration using the surrogate function is much smaller than computing the response using the original function (whose evaluation requires solving a system of ordinary differential equations). However, the computations associated with these techniques are still substantial, since they must employ a large number of samples to sufficiently reduce the Monte Carlo estimation error. By contrast, the techniques discussed in this paper are based on surrogate functions that lead to analytical formulas for the sensitivity indices, thus avoiding Monte Carlo estimation. As a matter of fact, the computational cost for calculating the variance-based sensitivity indices using the techniques discussed in this paper is mainly associated with the problem of estimating the underlying parameters of the surrogate function used, which leads to appreciable computational savings over the techniques proposed in [26-29].

## Methods

### Biochemical reaction systems

In this paper, we consider a well-stirred (homogeneous) biochemical reaction system at constant temperature and volume that consists of *M *coupled reactions of the form:

where *κ*_2*m*-1_, *κ*_2*m *_≥ 0 are the normalized rate constants of the forward and reverse reactions (measured in s^-1^) and *ν*_*nm*_,  ≥ 0 are the stoichiometry coefficients of the reactants and products. We assume that the system consists of *N *molecular species *X*_1_, *X*_2_, ..., *X*_*N*_, with concentrations (measured in molecules/cell) at time *t *≥ 0 given by *q*_1_(*t*), *q*_2_(*t*), ..., *q*_*N*_(*t*), respectively. We characterize the dynamic evolution of molecular concentrations by the following chemical kinetic equations:(1)

where  is the stoichiometry coefficient of the *n*^th ^molecular species associated with the *m*^th ^reaction and(2)

is the flux of the *m*^th ^reaction at time *t*.

**Figure 1:**
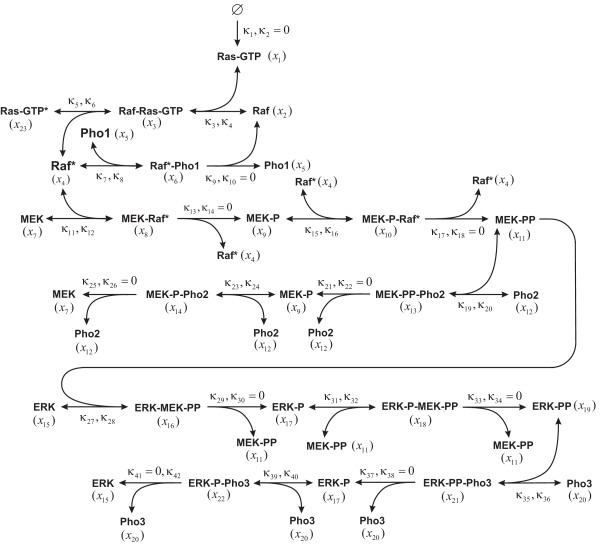
**A biochemical reaction model of the MAPK signaling cascade, adopted from Zhang *et al. ***[16].

The sensitivity analysis approach we consider here is based on quantifying the influence of a reaction or molecular species on an appropriately chosen response characteristic *R *of a biochemical reaction system. We employ a well-known model of the MAPK signaling cascade (see Figure 1 and Additional file 2 for details on this model) and consider three response characteristics with established biological significance, namely the *duration D*, *integrated response I*, and *strength S *of the doubly phosphorylated extracellular signal-regulated kinase (ERK-PP), defined by

where *q*(*t*) is the concentration profile of ERK-PP and *t*_0 _is the time at which *q*(*t*) converges to zero. If convergence to zero does not occur within the observation time interval [0, *t*_max_], then we set *t*_0 _= *t*_max_. We choose to work with the duration, integrated response, and strength of ERK-PP activity, since it has been experimentally observed that differences in duration and strength may cause distinct biological outcomes, such as cell differentiation, proliferation, and apoptosis [30-34], whereas, the integrated response directly correlates with DNA synthesis [35,36]. We take the system response *R *to be the logarithm of the duration, integrated response, or strength; that is, we take *R *to be ln*D*, ln*I*, or ln*S*. This reduces the effect of outliers and increases the efficiency of numerically evaluating the indices associated with variance-based sensitivity analysis [16].

### Variance-based sensitivity analysis

We employ the variance-based sensitivity analysis approach for biochemical reaction systems we recently introduced in [16]. This method is based on a biophysically-derived probabilistic model for the rate constants of a biochemical reaction system. According to this model, we treat the rate constants *κ*_2*m*-1 _and *κ*_2*m *_as random variables *K*_2*m*-1 _and *K*_2*m*_, given by the Eyring-Polanyi equations [37](3)

where *k*_*B *_is the Boltzmann constant (*k*_*B *_= 1.3806504 × 10^-23^JK^-1^), *T *is the system temperature, *h *is the Planck constant (*h *= 6.62606885 × 10^-34^Js),  is the (random) capacity of the activated complex associated with the *m*^th ^reaction, and *C*_*n *_is the (random) capacity of the *n*^th ^molecular species. The capacities are defined by(4)

where ,  are the (random) standard chemical potentials of the *m*^th ^activated complex and the *n*^th ^molecular species, respectively, given by(5)

In (5),  and  are the nominal standard chemical potential values associated with the *m*^th ^reaction and the *n*^th ^molecular species, whereas,  and *Y*_*n *_are zero-mean Gaussian random variables with standard deviations  and *λ*_*n*_, respectively. These random variables account for variations in the standard chemical potentials about their nominal values caused by unpredictable biological variability and uncertainty regarding their exact values. Similarly to [16], we assume that the random variables , *m *= 1, 2, ..., *M*, and *Y*_*n*_, *n *= 1, 2, ..., *N*, are statistically independent.

Our variance-based sensitivity analysis technique assesses how uncertainty in the rate constants of a biochemical reaction system affect the system response. As a consequence of (3), (4), and (5), we have that(6)

where

are the nominal values of the rate constants, with

Equation (6) suggests that uncertainty in the forward and reverse reaction rates occurs due to uncertainty in the standard chemical potentials of the activated complexes associated with the reactions and the standard chemical potentials of the reactants.

As a consequence of the previous model, we investigate the sensitivity properties of a biochemical reaction system due to the uncertainty in the standard chemical potentials. To simplify notation, we use ***W ***= {*W*_1_, *W*_2_, ..., *W*_*J*_} to denote the random variables *Y*^‡ ^and *Y*. We consider two cases, namely *J *= *M *and *W*_*j *_= , for *j *= 1, 2, ..., *M*, as well as *J *= *N *and *W*_*j *_= *Y*_*j*_, *j *= 1, 2, ..., *N*. In the first case, the standard chemical potentials of the molecular species are assumed to be fixed, whereas, the standard chemical potentials of the activated complexes are perturbed randomly. Our objective is to investigate the importance of reactions in influencing the system response and, for this reason, we refer to this case as *reaction-oriented sensitivity analysis *(ROSA) [16]. In the second case, the standard chemical potentials of the activated complexes are assumed to be fixed, whereas, the standard for chemical potentials of the molecular species are perturbed randomly. In this case, our objective is to investigate the importance of molecular species in influencing the system response. For this reason, we refer to this case as *species-oriented sensitivity analysis *(SOSA) [16].

Given the response *R *of a biochemical reaction system with random factors ***W***, its total variance *V*_tot _:= Var[*R*(***W ***)] satisfies [17,38,39]:(7)

where(8)

with similar expressions for the remaining terms. If the biochemical factors ***W ***are statistically independent (which we assume here to be true), then each term on the right-hand-side of (7) is nonnegative. This equation provides a decomposition of the total system response variance *V*_tot _into individual terms *V*_1_,*V*_2_, ..., *V*_12_, .... It turns out that *V*_*j *_quantifies the average reduction in total response variance, obtained by keeping the *j*^th ^biochemical factor fixed. As a consequence, we use *V*_*j *_to measure the *singular *influence of the *j*^th ^biochemical factor *W*_*j *_on the system response. Moreover, the term *V*_*jj' *_quantifies the average reduction in the total response variance due to jointly fixing the two biochemical factors *W*_*j *_and *W*_*j'*_, not accounted for by summing the average reductions obtained by separately fixing these factors. Therefore, we use *V*_*jj' *_to measure the *joint *influence of the biochemical factors *W*_*j *_and *W*_*j' *_on the system response. Finally, higher-order terms in (7) quantify the joint influence of three or more biochemical factors on the system response.

In most practical situations, it is difficult to evaluate the high-order terms (≥ 3) in the response variance decomposition scheme given by (7). Although these terms are usually negligible at low to moderate levels of biochemical factor uncertainty, they may take substantial values at high levels [16]. Unfortunately, it is difficult to deal in practice with high-order variance terms. For this reason, it is quite convenient to base our sensitivity analysis effort only on the first- and second-order terms *V*_*j *_and *V*_*jj'*_. Then, instead of using the total system response variance *V*_tot_, we base our sensitivity analysis on its second-order portion *V*, given by(9)

By using the probabilistic model given by (6) and the variance decomposition scheme in (9), we can develop a powerful (second-order) methodology for sensitivity analysis of biochemical reaction systems, similar to the one discussed in [16] that was based on the total response variance *V*_tot_. The method requires evaluation of two indices, namely the (second-order) *single-effect sensitivity index *(SESI) *σ*_*j*_, defined by(10)

and the (second-order) *joint-effect sensitivity index *(JESI) *η*_*j*_, defined by(11)

where(12)

Clearly, *σ*_*j *_quantifies the fractional singular contribution of the *j*^th ^biochemical factor to the second-order portion *V *of the total response variance, whereas, *η*_*j *_quantifies the fractional contribution of the *j*^th ^biochemical factor to *V *jointly with another factor. It turns out that, if *σ*_*j *_= *η*_*j *_= 0, then we can conclude that factor *j *does not influence the system response singularly or jointly with another factor (although, it may influence the system response jointly with two or more factors). On the other hand, if *σ*_*j *_> 0 and *η*_*j *_= 0, then we can conclude that factor *j *influences the system response singularly but not jointly with another factor. Moreover, if *σ*_*j *_= 0 and *η*_*j *_> 0, we can conclude that factor *j *does not influence the system response singularly but it does so jointly with some other factor, whereas, if *σ*_*j *_> 0 and *η*_*j *_> 0, we can conclude that factor *j *influences the system response both singularly and jointly with some other factor. In practice, we can set a small threshold *θ *to determine whether *σ*_*j *_and *η*_*j *_are sufficiently larger than zero.

Unfortunately, we cannot evaluate the exact values of the sensitivity indices *σ*_*j *_and *η*_*j*_. For this reason, we must resort to approximations. In this paper, we consider the possibility of employing one of five methods to accomplish this goal. We discuss these methods next and refer the reader to [16] and the accompanying Additional file 1 for details pertaining to their development and numerical implementation.

### Monte Carlo estimation

A straightforward technique for approximating the SESI and JESI values is based on a Monte Carlo Latin hypercube sampling approach, whose details can be found in [16] (see also [2,20]). This approach can be used to provide estimates  and  of the second-order SESI's and JESI's by using 2*L*(*J *+ 1) system evaluations [by integrating the system of *N *ordinary differential equations given by (1) and (2)], where *L *is the number of Latin hypercube samples used and *J *is the number of biochemical factors considered in the analysis. We refer to  and  as the (second-order) SESI's and JESI's obtained by *Monte Carlo *(MC) estimation. This method is computationally expensive, since a large number *L *of Latin hypercube samples is required to obtain sufficiently accurate estimates of the sensitivity indices.

### Derivative approximation

A method for deriving approximations  and  of the sensitivity indices *σ*_*j *_and *η*_*j *_is to replace the response function *R*(***w***) by its second-order Taylor series approximation  about ***w ***= **0**, given by(13)

where

are the first- and second-order partial derivatives of *R *at ***w ***= **0**, and set(14)

where(15)

Equation (13) and the statistical independence and zero-mean Gaussianity of the biochemical factors *W*_*j *_imply that(16)

where *λ*_*j *_is the standard deviation of *W*_*j*_, for *j *= 1, 2, ..., *J*. As a consequence, we obtain an analytical expression for the sensitivity indices  and , which requires evaluation of the first- and second-order partial derivatives of the response function *R*(***w***), with respect to the biochemical factors, at ***w ***= **0**.

Although many techniques have been developed to compute response derivatives [40], for reasons we explain in Additional file 1, we choose to approximate the partial derivatives by symmetric finite differences. We refer to  and  given by (14), (15), and (16), as the (second-order) SESI's and JESI's obtained by *Derivative Approximation *(DA). The resulting method requires 2*J*(*J *+ 1) + 1 system integrations, which is quadratic in terms of the number *J *of the biochemical factors and is much smaller than the number 2*L*(*J *+ 1) of system integrations required by MC, since *J *« *L*.

### Polynomial approximation

Another way to approximate the sensitivity indices *σ*_*j *_and *η*_*j *_is to replace the response function *R*(***w***) by(17)

where the *α*'s are parameters whose values must be appropriately determined so that (***w***) sufficiently approximates the response function *R*(***w***) in an appropriately chosen neighborhood around **0**. Note that  provides a polynomial approximation of the response function *R*(***w***). If  is sufficiently close to *R*(***w***) in a neighborhood around **0**, then the parameters *α_jj'_*coincide with the partial derivatives , 1 ≤ *κ*_1_, *κ*_2 _≤ 2, of *R *at ***w *= 0**.

By using (17) and the statistical independence and zero-mean Gaussianity of the biochemical factors *W*_*j*_, we can show that, in this case,  and  are given by (14) and (15), with(18)

As a consequence, we obtain again an analytical expression for the sensitivity indices  and , which requires evaluation of the *α *parameters. This can be done by the polynomial regression approach we discuss in Additional file 1. We refer to  and , given by (14), (15), and (18), as the (second-order) SESI's and JESI's obtained by *Polynomial Approximation *(PA). The resulting method is based on the approach proposed in [41] and requires *J*(*J - *1)*S*^2^/2 + *JS *+ 1 system integrations, which is quadratic both in terms of the number *J *of biochemical factors and the number *S *of the samples per factor used in the regression. Note that *J*(*J *- 1)*S*^2^/2 + *JS *+ 1 ≃ 2*J*^2^(*S*/2)^2^, for sufficiently large *J*. This number is much smaller than the number 2*L*(*J *+ 1) ≃ 2*LJ *of system integrations required by MC, since *L *≫ *J*(*S*/2)^2^, but larger than the number 2*J*(*J *+ 1) + 1 ≃ 2*J*^2 ^of system integrations required by DA, since *S *> 2.

### Gauss-Hermite integration

We can obtain a more accurate approximation  of the response function *R*(***w***) than the one given by (13) if we truncate the Taylor series expansion of *R*(***w***) about ***w ***= **0 **by removing all terms that involve partial derivatives with respect to more than two factors [note that the approximation given by (13) is obtained from the Taylor series expansion by truncating all terms that involve partial derivatives of order greater than two]. In this case, we can show that

where(19)

as we explain in Additional file 1. The approximations  and  are now given by (14) and (15), with(20)

where *e*_0_, *e*_*j*_, and *e*_*jj' *_are given by(21)

Note that evaluation of  and  requires only one- and two-dimensional integrations, which can be numerically done by a standard Gauss-Hermite integration approach. For this reason, we refer to  and , given by (14), (15), (19), (20), and (21), as the (second-order) SESI's and JESI's obtained by *Gauss Hermite Integration *(GHI). The resulting method is based on the approach proposed in [42,43] and requires 2*J*(*J *- 1)⌊*Q*/2⌋^2 ^+ 2*J*⌊*Q*/2⌋ + 1 system integrations, which is quadratic both in terms of the number *J *of biochemical factors and the order *Q *of Gauss-Hermite integration used. Note that, if the number *S *of the samples per factor used in the regression associated with the PA is even, and *Q *= *S *or *Q *= *S *+ 1, then GHI requires the same number of system integrations as PA.

### Orthonormal Hermite approximation

The last method we consider for approximating the sensitivity indices *σ*_*j *_and *η*_*j *_is based on replacing the response function *R*(***w***) by(22)

where the *α*'s and  are parameters whose values must be appropriately determined so that  sufficiently approximates the response function *R*(***w***) over the entire space of biochemical factor values. Note that  provides a polynomial approximation of the response function *R*(***w***), similar to the one given by (17). However, the polynomials used in the approximation given by (22) are orthonormal Hermite polynomials, as opposed to the polynomials used in the approximation given by (17), which are standard second- and fourth-order polynomials. Note also that the approximation given by (22) is "global," in the sense that it is based on approximating the system response function *R*(***w***) over the entire factor space, whereas, the approximation given by (17) is "local," in the sense that it approximates the system response function *R*(***w***) in a neighborhood around ***w ***= **0**.

By using (22), the orthonormality of the Hermite polynomials, and the statistical independence and zero-mean Gaussianity of the biochemical factors *W*_*j*_, we can show that  and  are given by (14) and (15), with(23)

As a consequence, we obtain again an analytical expression for the sensitivity indices  and , which requires evaluation of the *α *parameters. This can be done by polynomial regression based on the Monte Carlo Latin hypercube sampling approach we discuss in Additional file 1. We refer to  and , given by (14), (15), and (23), as the (second-order) SESI's and JESI's obtained by *Orthonormal Hermite Approximation *(OHA). The resulting method is based on the approach suggested in [44-47] and requires *L *system integrations, where *L *is the number of regression points obtained by Latin hypercube sampling. We here take the number of regression points used to be the same as the number of Latin hypercube samples employed by MC, although these two numbers can be different in general. As a consequence, the number of system integrations performed by OHA is smaller than the number 2*L*(*J *+ 1) of system integrations used in MC by a factor of 2(*J *+ 1), but it could be larger than the number of system integrations required by DA, PA, or GHI.

## Results

We now employ the previously discussed techniques to estimate the variance-based sensitivity indices *σ*_*j *_and *η*_*j *_associated with the duration, integrated response, and strength of ERK-PP activity. We do this by considering the dynamic behavior, within a time frame of 6 hours, of the MAPK signaling cascade model depicted in Figure 1 (see Additional file 2 for more details on this model). As we have explained in the previous section, we consider two cases: ROSA and SOSA. In each case, we need to set values for the standard deviations {, *m *= 1,2, ..., *M*} of the standard chemical potentials of the activated complexes of the reactions and the standard deviations {*λ*_*n*_, *n *= 1,2, ..., *N*} of the standard chemical potentials of the molecular species. Due to difficulties in obtaining these values in practice, we assume here that  = *λ*^‡^, for *m *= 1,2, ..., *M*, and *λ*_*n *_= *λ*, for *n *= 1,2, ..., *N*, and consider *λ*^‡^, *λ *as two "user-defined" parameters that quantify the perturbation levels in biochemical factor values. By following our previous work in [16], we perform sensitivity analysis with *λ*^‡^, *λ *= 0.1, 0.2, 0.3, 0.4. Finally, we employ *L *= 6,000 Latin hypercube samples in MC and OHA, *S *= 4 regression samples per factor in PA, and a Gauss-Hermite integration of order *Q *= 5 in GHI.

In our simulations, we use *S *= 4 regression points per biochemical factor, located at -2*w*, -*w*, *w*, and 2*w*, where *w *= *λ*^‡ ^for ROSA and *w *= *λ *for SOSA (i.e., we use regression points located at ± one and two standard deviations from **0**). Note also that, as a consequence of equation (6), if *Y*_*n *_= 0, for *n *= 1, 2, ..., *N*, then a ± *λ*^‡ ^variation in the values of  about zero will produce a variation in the nominal values of the rate constants of the *m*^th ^reaction within the percentage interval 100[exp{-} - 1, exp{} - 1]%. This corresponds to variations in the nominal values of the reaction rate constants within the interval [-9.52%, 10.52%], for *λ*^‡ ^= 0.1, [-18.13%, 22.14%], for *λ*^‡ ^= 0.2, [-25.92%, 34.99%], for *λ*^‡ ^= 0.3, and [-32.97%, 49.18%], for *λ*^‡ ^= 0.4.

In Table 1, we summarize the number of system integrations and the equations used by each method. For ROSA-based sensitivity analysis (*J *= 21), the number of system integrations required by DA, PA, GHI, and OHA, are respectively only 0.35%, 1.30%, 1.30%, and 2.27% of that required by MC. For SOSA-based sensitivity analysis (*J *= 23), the number of system integrations required by DA, PA, GHI, and OHA, are respectively only 0.38%, 1.44%, 1.44%, and 2.08% of that required by MC.

**Table 1:** Required system integrations, equations used, and sources of error.

Method	System Integrations	ROSA	SOSA	Equations Used	Error Sources
**MC**	2*L*(*J *+ 1)	264000	288000	(10)-(12)	• number of MC samples used
**DA**	2*J*(*J *+ 1) + 1	925	1105	(14)-(16)	• local approximation
					• truncation of Taylor series
					• derivative approximation
**PA**	*J*(*J *- 1)*S*^2^/2 + *JS *+ 1	3445	4141	(14), (15), (18)	• local approximation
					• truncation of FD-HDMR
					• polynomial approximation
					• polynomial regression
**GHI**	2*J*(*J *- 1)⌊*Q*/2⌋^2 ^+ 2*J*⌊*Q*/2⌋ + 1	3445	4141	(14), (15), (19)-(21)	• local approximation
					• truncation of FD-HDMR
					• Gauss-Hermite integration
**OHA**	*L*	6000	6000	(14), (15), (23)	• truncation of ANOVA-HDMR
					• Hermite approximation
					• polynomial regression

*L*: number of Monte Carlo (Latin hypercube) samples.

*J*: number of biochemical factors.

*S*: number of regression samples per factor.

*Q*: order of Gauss-Hermite integration.

We list the ROSA results in Tables 2, 3, and 4, whereas, we list the SOSA results in Tables S-3.1, S-3.2, and S-3.3 of Additional file 3. The results are given in percentages and have been truncated to the nearest integers. To reduce the size of the tables, we depict only the results associated with reactions whose truncated SESI or JESI values, estimated by MC, are at least 5%. We consider the SESI and JESI values obtained by MC as the "true" values. By following our previous work in [16], we classify reactions and molecular species into one of four categories of interest: singularly influential, jointly influential, singularly/jointly influential, and noninfluential. We do this by comparing their SESI and JESI values to a 10% threshold. Bold reaction numbers indicate SESI or JESI values, obtained by MC, that are above the 10% threshold. Note that a reaction is singularly influential if the corresponding SESI value is at least 10% and the JESI value is smaller than 10%, jointly influential if the JESI value is at least 10% and the SESI value is smaller than 10%, singularly/jointly influential if both the SESI and JESI values are at least 10%, and noninfluential if both the SESI and JESI values are smaller than 10%.

**Table 2:** ROSA-based sensitivity analysis results for the *duration *of ERK-PP activity.

SESI - DURATION (*λ*^‡ ^= 0.1)						JESI - DURATION (*λ*^‡ ^= 0.1)					
**Reaction**	**MC**	**DA**	**PA**	**GHI**	**OHA**	**Reaction**	**MC**	**DA**	**PA**	**GHI**	**OHA**

**4**	28	28	28	27	28	4	1	0	0	0	0
**6**	24	26	25	22	25	6	1	0	0	0	0
11	7	7	7	9	8	11	0	0	0	0	0
**13**	18	18	20	18	19	13	1	0	0	0	0

**SESI - DURATION (*λ*^‡ ^= 0.2)**						**JESI - DURATION (*λ*^‡ ^= 0.2)**					

**Reaction**	**MC**	**DA**	**PA**	**GHI**	**OHA**	**Reaction**	**MC**	**DA**	**PA**	**GHI**	**OHA**

**4**	26	27	27	29	27	4	2	1	1	1	1
**6**	22	25	25	25	23	6	2	1	1	1	1
11	7	7	7	8	8	11	1	0	0	0	0
**13**	16	17	18	16	17	13	1	1	0	0	0
17	5	5	6	4	5	17	1	1	1	1	1
21	5	5	5	6	5	21	1	1	0	1	1

**SESI - DURATION (*λ*^‡ ^= 0.3)**						**JESI - DURATION (*λ*^‡ ^= 0.3)**					

**Reaction**	**MC**	**DA**	**PA**	**GHI**	**OHA**	**Reaction**	**MC**	**DA**	**PA**	**GHI**	**OHA**

**4**	26	26	26	24	26	4	1	2	2	2	2
**6**	21	24	20	21	21	6	1	2	1	1	1
11	7	6	7	7	8	11	0	1	0	0	0
**13**	15	16	13	15	15	13	1	1	1	1	1
17	5	4	6	5	5	17	1	2	2	2	1
21	6	5	8	8	6	21	2	2	3	2	1

**SESI - DURATION (*λ*^‡ ^= 0.4)**						**JESI - DURATION (*λ*^‡ ^= 0.4)**					

**Reaction**	**MC**	**DA**	**PA**	**GHI**	**OHA**	**Reaction**	**MC**	**DA**	**PA**	**GHI**	**OHA**

**4**	23	24	23	21	25	4	4	3	2	3	3
**6**	19	22	20	19	21	6	4	3	2	2	2
11	8	6	6	7	9	11	1	1	0	0	0
**13**	14	15	12	11	15	13	1	2	1	1	1
17	5	4	6	8	5	17	2	3	2	3	1

**Table 3:** ROSA-based sensitivity analysis results for the *integrated response *of ERK-PP activity.

SESI - I-RESPONSE (*λ*^‡ ^= 0.1)						JESI - I-RESPONSE (*λ*^‡ ^= 0.1)					
**Reaction**	**MC**	**DA**	**PA**	**GHI**	**OHA**	**Reaction**	**MC**	**DA**	**PA**	**GHI**	**OHA**

**4**	39	39	39	39	39	4	1	0	0	0	0
**6**	26	27	27	27	27	6	1	0	0	0	0
11	9	10	9	9	9	11	0	0	0	0	0
13	8	8	8	8	8	13	0	0	0	0	0

**SESI - I-RESPONSE (*λ*^‡ ^= 0.2)**						**JESI - I-RESPONSE (*λ*^‡ ^= 0.2)**					

**Reaction**	**MC**	**DA**	**PA**	**GHI**	**OHA**	**Reaction**	**MC**	**DA**	**PA**	**GHI**	**OHA**

**4**	37	38	40	40	39	4	5	1	1	2	2
**6**	25	27	26	26	25	6	4	0	0	1	1
8	5	5	5	5	6	8	2	0	0	1	1
11	7	9	8	8	8	11	1	0	0	0	0
13	6	8	7	7	7	13	1	1	0	0	0

**SESI - I-RESPONSE (*λ*^‡ ^= 0.3)**						**JESI - I-RESPONSE (*λ*^‡ ^= 0.3)**					

**Reaction**	**MC**	**DA**	**PA**	**GHI**	**OHA**	**Reaction**	**MC**	**DA**	**PA**	**GHI**	**OHA**

**4**	38	37	43	41	36	**4**	10	2	9	10	11
**6**	21	26	22	21	21	6	7	1	4	4	6
8	8	4	7	7	7	8	4	0	3	4	5

**SESI - I-RESPONSE (*λ*^‡ ^= 0.4)**						**JESI - I-RESPONSE (*λ*^‡ ^= 0.4)**					

**Reaction**	**MC**	**DA**	**PA**	**GHI**	**OHA**	**Reaction**	**MC**	**DA**	**PA**	**GHI**	**OHA**

**4**	36	36	43	40	34	**4**	15	3	18	15	16
**6**	18	25	16	19	18	6	8	2	7	7	8
8	8	4	8	9	8	8	7	1	6	6	7

**Table 4:** ROSA-based sensitivity analysis results for the *strength *of ERK-PP activity.

SESI - STRENGTH (*λ*^‡ ^= 0.1)						JESI - STRENGTH (*λ*^‡ ^= 0.1)					
**Reaction**	**MC**	**DA**	**PA**	**GHI**	**OHA**	**Reaction**	**MC**	**DA**	**PA**	**GHI**	**OHA**

**4**	38	38	36	30	38	4	1	0	0	0	0
**6**	17	15	15	14	17	6	1	1	0	0	0
**8**	10	10	9	6	10	8	1	0	0	0	0
11	8	9	9	4	8	11	0	0	0	0	0
**19**	12	10	12	15	13	19	1	1	0	0	0

**SESI - STRENGTH (*λ*^‡ ^= 0.2)**						**JESI - STRENGTH (*λ*^‡ ^= 0.2)**					

**Reaction**	**MC**	**DA**	**PA**	**GHI**	**OHA**	**Reaction**	**MC**	**DA**	**PA**	**GHI**	**OHA**

**4**	32	34	40	39	33	**4**	13	2	3	8	11
**6**	14	14	14	12	13	6	8	3	1	3	6
8	8	9	11	12	9	8	7	1	1	2	5
17	6	4	6	3	6	17	6	1	1	2	4
**19**	10	9	11	12	12	19	5	2	1	1	4

**SESI - STRENGTH (*λ*^‡ ^= 0.3)**						**JESI - STRENGTH (*λ*^‡ ^= 0.3)**					

**Reaction**	**MC**	**DA**	**PA**	**GHI**	**OHA**	**Reaction**	**MC**	**DA**	**PA**	**GHI**	**OHA**

**4**	31	30	37	37	27	**4**	23	3	22	25	26
**6**	10	12	12	11	10	**6**	17	5	9	10	15
8	9	8	10	9	8	**8**	11	2	8	9	11
19	6	8	7	6	5	19	5	4	3	3	4

**SESI - STRENGTH (*λ*^‡ ^= 0.4)**						**JESI - STRENGTH (*λ*^‡ ^= 0.4)**					

**Reaction**	**MC**	**DA**	**PA**	**GHI**	**OHA**	**Reaction**	**MC**	**DA**	**PA**	**GHI**	**OHA**

**4**	28	25	40	36	26	**4**	28	5	29	27	29
5	2	1	1	0	2	5	6	5	2	2	5
**6**	10	10	9	11	10	**6**	16	7	11	11	15
8	8	7	8	10	8	**8**	15	3	11	11	14
15	1	0	0	0	2	15	7	5	4	4	7
21	1	0	0	0	1	21	7	4	4	4	8

In the remaining of this section, we discuss the ROSA results separately for each response characteristic. A similar discussion applies for the SOSA results presented in Additional file 3.

### Duration

Estimation, by MC, of the ROSA-based sensitivity indices associated with the duration of ERK-PP activity produces values that change little with the size *λ*^‡ ^of the underlying perturbations; see Table 2. Moreover, the estimated SESI and JESI values indicate that the duration is primarily affected by reactions 4, 6, and 13 (refer to Figure 1 and Additional file 2 for identifying these reactions), which exert their influence only singularly (since the SESI values are larger than 10%, whereas the corresponding JESI values are less than 10%). As a matter of fact, all JESI values are negligible, which indicates that the log-duration may be approximately additive, at least within the range of the applied perturbations. Note that a multivariate response function is called additive if it can be decomposed into a sum of one-dimensional functions of one variable. Additive response functions do not produce high-order (≥ 2) joint effects and result in zero JESI values [2]. Although a linear response function is additive, the inverse is not necessarily true. It turns out that the SESI's associated with an additive response function can be well estimated by all previous approximation techniques.

From the results depicted in Table 2 (and Table S-3.1 in Additional file 3), it is clear that, as compared to MC, the DA, PA, GHI, and OHA consistently provide good approximations to the SESI and JESI values at all perturbation levels. Moreover, all methods can be used to correctly classify reactions 4, 6, and 13 as being singularly influential.

### Integrated response

Estimation, by MC, of the ROSA-based sensitivity indices associated with the integrated response of ERK-PP activity produces the SESI and JESI values depicted in Table 3. These values indicate that the integrated response is primary influenced by reactions 4 and 6 (refer to Figure 1 and Additional file 2 for identifying these reactions). For small to moderate perturbations (i.e., for *λ*^‡ ^= 0.1, 0.2), reactions 4 and 6 influence the integrated response only singularly. However, for large perturbations (i.e., for *λ*^‡ ^= 0.3, 0.4), reaction 4 influences the integrated response both singularly and jointly (since both SESI and JESI values are at least 10%), whereas, reaction 6 still influences the integrated response only singularly.

It is clear from the results depicted in Table 3 (and Table S-3.2 in Additional file 3) that all approximation techniques work relatively well for small to moderate perturbation levels (i.e., for *λ*^‡ ^= 0.1, 0.2), providing accurate SESI and JESI values, as compared to the values obtained by MC, and produce correct classification of the reactions. This is true, since the log integrated response may be approximately additive in this case, as indicated by the negligible JESI values. However, for large perturbations (i.e., for *λ*^‡ ^= 0.3, 0.4), the log integrated response is not additive anymore and the results obtained by DA deteriorate noticeably, deeming the use of DA inappropriate. For example, using the JESI results produced by ROSA, the largest differences between the values obtained by DA and MC are 8% and 12% for *λ*^‡ ^= 0.3, 0.4, respectively. As a matter of fact, the DA is not capable of capturing second-order joint effects and the resulting JESI values are very small. If we use the DA results to classify the reactions, then we will erroneously conclude that reaction 4 influences the integrated response only singularly, when *λ*^‡ ^= 0.3, 0.4.

From the results depicted in Table 3 (and Table S-3.2 in Additional file 3), it is clear that, for large perturbations, GHI and OHA provide good approximations to the sensitivity indices. Moreover, the results indicate that OHA may be a better approximation technique than GHI (e.g., compare the SESI results obtained by GHI and OHA for reaction 4). On the other hand, the results obtained by PA are much better than the results obtained by DA. However, the performance of PA may deteriorate at high perturbation levels and may become inferior to GHI and OHA (e.g., compare the results obtained by PA, GHI, and OHA for reaction 4). Finally, it is clear that the sensitivity results obtained by GHI and OHA can be used to correctly classify all reactions.

### Strength

Estimation, by MC, of the ROSA-based sensitivity indices associated with the strength of ERK-PP activity produces the SESI and JESI values depicted in Table 4. These values indicate that the log strength may be approximately additive when *λ*^‡ ^= 0.1. However, the log strength becomes nonadditive when *λ*^‡ ^= 0.2, 0.3, 0.4, since the estimated JESI values are not negligible at these perturbation levels. Note that, when *λ*^‡ ^= 0.1, the strength is primarily affected by reactions 4, 6, 8, and 19, which exert their influence only singularly. However, when *λ*^‡ ^= 0.2, reaction 8 becomes noninfluential, reaction 4 influences the strength both singularly and jointly, whereas, reactions 6 and 19 still influence the strength singularly. On the other hand, when *λ*^‡ ^= 0.3, 0.4, reactions 4 and 6 influence the strength both singularly and jointly, whereas, reaction 8 influences the strength only jointly (since the JESI values are larger than 10%, whereas, the corresponding SESI values are less than 10%).

It is clear from the results depicted in Table 4 (and Table S-3.3 in Additional file 3) that all approximation techniques work relatively well when *λ*^‡ ^= 0.1, producing accurate SESI and JESI values, as compared to the values obtained by MC, and resulting in correct classification of the reactions. However, when *λ*^‡ ^= 0.2, 0.3, 0.4, DA produces inaccurate results, while the performance of PA and GHI deteriorates noticeably. For example, using the JESI results produced by ROSA, the largest differences between the values obtained by DA and MC are 11%, 20% and 23% for *λ*^‡ ^= 0.2, 0.3, 0.4, respectively. Moreover, the largest differences between the values obtained by PA and MC are 10%, 8% and 5% for *λ*^‡ ^= 0.2, 0.3, 0.4, respectively. Finally, the largest differences between the values obtained by GHI and MC are 5%, 7% and 5% for *λ*^‡ ^= 0.2, 0.3, 0.4, respectively. Once more, OHA consistently provides good results, which can be used to correctly classify the reactions at all perturbation levels.

## Discussion

The previous numerical results demonstrate that, in terms of estimation accuracy, OHA is the best method and DA is the worst, whereas, PA and GHI are in between, with GHI slightly better than PA. To explain why this is so, we must investigate the sources of error introduced by each technique, which we summarize in Table 1.

The estimation error produced by the MC approach is mainly due to the finite number *L *of samples used and decreases slowly as *L *increases, regardless of the number *J *of biochemical factors used, at least theoretically. Note, however, that to achieve a certain level of accuracy in practice, we may also need to increase *L *as the number *J *of biochemical factors increases, due to the exponential growth in the volume of the biochemical factor space when adding extra dimensions ("curse of dimensionality").

There are two sources of error associated with DA. First, substantial errors may be introduced due to the fact that DA *locally *approximates the response function by a Taylor series expansion that includes only first- and second-order partial derivatives. Consequently, DA may not produce good estimates of the sensitivity indices under large perturbations, since a second-order Taylor series approximation of the response function may not be sufficiently accurate over the range of factor values generated by such perturbations. This is especially true when the response function is nonadditive (as it is the case with the log integrated response and the log strength of ERK-PP in the MAPK example). In such cases, large factor variations may produce substantial joint effects, which cannot be captured by a local second-order Taylor series approximation. This is evident by the fact that, under large perturbations, the JESI values obtained by DA, associated with the integrated response and strength, are significantly different than the ones produced by MC.

A second source of error associated with DA is the approximation of the first- and second-order derivatives of the response function by finite-differences. In our simulations, we approximate the first- and second-order partial derivatives of the response function by using equations (S-1.35) and (S-1.36) in Additional file 1, with Δ = 0.1. It has been pointed out in [1] that the resulting approximations must be carefully used, since it is difficult to theoretically predict, control, and numerically evaluate their accuracy. Although a number of techniques have been developed to deal with this problem [40], exact evaluation of the response derivatives usually requires simultaneous integration of a set of "sensitivity equations," together with the differential equations governing the underlying molecular concentration dynamics, which turns out to be a very difficult task due to stiffness of the resulting system of differential equations [1].

PA attempts to improve the accuracy of DA by adding high-order derivative terms in the Taylor series expansion of the response function. In addition to the first- and second-order partial derivatives used by the DA, the Taylor series expansion now includes third- and fourth-order partial derivatives that involve only two biochemical factors. Moreover, instead of approximating the derivatives by finite differences, the method avoids such computations by expanding the response function using FD-HDMR, by truncating all components of order ≥ 3, by respectively approximating the first- and second-order FD-HDMR components with second- and fourth-order polynomials, and by estimating the coefficients of these polynomials using regression (see Additional file 1 for details). Errors are introduced by truncating the FD-HDMR and *locally *approximating the resulting response function by a fourth-order polynomial including only single biochemical factors and pairs of factors. As a consequence, PA may not be able to accurately estimate some SESI and JESI values under large perturbations, since the underlying truncation and polynomial approximation of the response function may not be sufficiently accurate over the range of factor values generated by such perturbations. Note also that errors can be introduced due to estimating the polynomial coefficients by regression, a situation that cannot be evaluated and controlled easily. As a matter of fact, and counter to intuition, we cannot necessarily increase accuracy of estimation by using more samples per biochemical factor, especially when dealing with polynomial regression [48,49].

GHI attempts to improve the accuracy of estimating the sensitivity indices by employing the *exact *first- and second-order FD-HDMR components, and numerically calculating the required expectations and variances using Gauss-Hermite integrations (see Additional file 1 for details). Errors are introduced when truncating the FD-HDMR and evaluating the expectations and variances by one- and two-dimensional Gauss-Hermite integrations. Evaluating and controlling these errors is practically impossible. Note that higher-order Gauss-Hermite integrations do not necessarily produce higher accuracy. This is true only when the integrands are sufficiently smooth, in the sense that can be well-approximated by polynomials [49]. Truncation of the FD-HDMR essentially corresponds to a *local *approximation of the response function, although this approximation is expected to be more accurate than the Taylor series and polynomial approximations used by DA and PA, respectively. As a consequence, GHI may not be able to accurately estimate some SESI and JESI values under large perturbations, since the underlying FD-HDMR truncation may not be sufficiently accurate over the range of factor values generated by such perturbations.

Finally, the errors introduced by OHA are due to approximating the ANOVA-HDMR expansion of the response function by first- and second-order ANOVA-HDMR components, approximating these components with first- and second-order orthonormal Hermite polynomials, and estimating the coefficients of these polynomials using regression (see Additional file 1 for details). Here, the truncation of high-order ANOVA-HDMR components does not correspond to a local approximation of the response function, which is why this approximation is more accurate than truncating the FD-HDMR components, as in GHI. In fact, if we consider perturbation levels at which the higher-order (≥ 3) terms in the variance decomposition scheme given by (7) are negligible, then the higher-order (≥ 3) terms in the ANOVA-HDMR decomposition of the response function will be negligible as well [see equation (S-1.30) in Additional file 1]. This is not necessarily true for the higher-order terms in the FD-HDMR decomposition. Therefore, truncating the ANOVA-HDMR decomposition of the response function, as opposed to the FD-HDMR decomposition, is well justified for perturbation levels at which the response variance is not appreciably influenced by high-order joint effects. Under very large perturbations, OHA may not accurately estimate the sensitivity indices, since the underlying truncation of ANOVA-HDMR may not be accurate enough due to appreciable high-order (≥ 3) joint effects in the response variance. However, the *global *nature of the approximation methodology employed by OHA, the direct relationship between ANOVA-HDMR and the response variance decomposition scheme given by (7), and the orthonormality properties of the Hermite polynomials, make OHA the most desirable technique for approximating the sensitivity indices, among the techniques considered in this paper.

Although we have also obtained simulation results for other biochemical reaction systems, due to lack of space, we have limited our presentation in this paper to the results obtained for the MAPK model depicted in Figure 1. To illustrate various aspects of the approximation techniques and their relative merits, we have chosen the response functions to represent three types of high-dimensional system responses: the log duration, ln*D*, is approximately additive for the levels of biochemical factor uncertainty considered in this paper, the log integrated response, ln*I*, is moderately nonadditive, whereas, the log strength, ln*S*, is highly nonadditive. Based on our experience so far, all our simulation results are consistent with each other and perfectly agree with the theoretical analysis presented in this paper. We therefore believe that the conclusions based on the MAPK model are general and can be applied to other biochemical reaction systems as well.

It is very important to keep in mind that the four approximation techniques considered in this paper are based on the assumption that, for most biochemical reaction systems of interest, perturbations of input biochemical factors will produce only single and second-order joint effects at the output. As a consequence, truncating the HDMR of the response function to a second-order is a natural thing to do. Note that this assumption depends on the particular choice of the biochemical factors used, on how the system response relates to these factors, and on the perturbation levels used for sensitivity analysis. In general, the approximation methods discussed in this paper are expected to fail in the presence of high-order ≥ 3 joint effects among biochemical factors. Therefore, it may be necessary in these cases to consider truncated HDMR's that include higher-order basis functions. Extension to this case is straightforward but computationally demanding, since higher-order cases require evaluation of a large number of variance terms in the decomposition scheme given by (7), which can be a tedious thing to do for large biochemical reaction systems.

We should point out here that GHI is based on the methodology proposed in [42,43], which has been effectively used to calculate statistical moments of the responses of high-dimensional mechanical systems subject to randomly fluctuating loads. In this paper, we have reformulated this method to fit the framework of variance-based sensitivity analysis and have applied it to biochemical reaction systems. On the other hand, OHA is based on the methodology proposed in [25,44,45,50] for approximating ANOVA-HDMR's using orthonormal basis functions. OHA can also be viewed as a special case of the polynomial chaos expansion (PCE) approach to sensitivity analysis discussed in [46,47,51], and has been recently employed in [52] for estimating variance-based sensitivity indices in order to learn the topology of a functional network of interactions from given data. To our knowledge, this is the first time that the four approximation techniques presented in this paper are systematically compared to each other and used to study the sensitivity properties of biochemical reaction systems.

To conclude, we would like to stress the fact that the approximation techniques presented in this paper have been derived by assuming that the biochemical factors used for sensitivity analysis are statistically independent and that each factor follows a Gaussian distribution. The assumption of statistical independence between the random variables {, *m *= 1,2, ..., *M*} and {*Y*_*n*_, *n *= 1, 2, ..., *N*} has been justified in [16]. However, justifying mutual independence within the sets {, *m *= 1, 2, ..., *M} *and {*Y*_*n*_, *n *= 1,2, ..., *N*} is a very difficult thing to do. We simply view this assumption as a convenient approximation that allows us to proceed with the sensitivity analysis approaches discussed in this paper. Developing variance-based sensitivity analysis for correlated biochemical factors is a challenging problem that needs careful investigation [2,53]. On the other hand, if the biochemical factors follow non-Gaussian distributions, such as uniform, gamma, binomial, etc., the approximation techniques must be appropriately modified to accommodate these distributions. For example, if each biochemical factor follows a uniform distribution, then we must replace the Gauss-Hermite integration step in GHI by Gauss-Legendre integration [49]. Moreover, if the biochemical factors follow gamma distributions, then we must replace the orthonormal Hermite polynomials in OHA by orthonormal Laguerre polynomials [47,51].

## Conclusions

In this paper, we discussed four methods that one can use to analytically approximate the second-order sensitivity indices associated with a previously introduced variance-based sensitivity analysis methodology for biochemical reaction systems. The need for developing such methods stems from an effort to remedy the large computational burden associated with Monte Carlo estimation. We highlighted important theoretical, numerical, and computational aspects of each method, in an attempt to provide a comprehensive understanding of the advantages and disadvantages of each technique. Our simulation results, based on a mathematical model for the MAPK signalling cascade, clearly demonstrate the inferiority of second-order derivative-based sensitivity analysis at moderate to high levels of uncertainty. It also shows the superiority of OHA, which is constructed by truncating the ANOVA-HDMR of the response function of a biochemical reaction system and approximating the first- and second-order ANOVA-HDMR component functions with orthonormal Hermite polynomials.

## Competing interests

The authors declare that they have no competing interests.

## Authors' contributions

JG performed the design of the study and drafted the manuscript. HXZ contributed significantly to the manuscript by coding all methods in Matlab, by acquiring data for the study, and by interpreting the results. Both authors read and approved the final manuscript.

## Supplementary Material

Additional file 1 **Approximation methods and implementations**. This file contains the mathematical details associated with the four approximation methods presented in the paper and discusses their numerical implementation. Click here for file

Additional file 2 **MAPK signaling cascade model**. This file lists the biochemical reactions associated with the MAPK signaling cascade model and provides nominal values for the normalized reaction rate constants and initial molecular concentrations. Click here for file

Additional file 3 **SOSA-based sensitivity analysis results**. This file summarizes the SOSA-based sensitivity analysis results for the three response characteristics (duration, integrated response, and strength) of ERK-PP activity in the MAPK signaling cascade obtained by the five approximation methods discussed in the paper. Click here for file
