# Supplementary material for: A comparison of approximation techniques for variance-based sensitivity analysis of biochemical reaction systems
Source: BMC Bioinformatics. 2010 May 12;11:246. doi: 10.1186/1471-2105-11-246 (PMC2894038; doi:10.1186/1471-2105-11-246)
Supplement: Additional file 1 — Approximation methods and implementations. This file contains the mathematical details associated with the four approximation methods presented in the paper and discusses their numerical implementation. [file 1471-2105-11-246-S1.PDF]

ADDITIONAL FILE 1

A comparison of approximation techniques for variance-based sensitivity analysis of biochemical reaction systems

APPROXIMATION METHODS AND IMPLEMENTATIONS

Hong-Xuan Zhang[1] **and John Goutsias***[1]

[1] Whitaker Biomedical Engineering Institute, The Johns Hopkins University, Baltimore, MD 21218, USA

* Corresponding author

Email: HXZ: hxzhang@jhu.edu, JG: goutsias@jhu.edu

In this document, we present several methods for approximating the indices σ_j and η_j associated with the second-order variance-based sensitivity analysis technique discussed in the Main text. We first review a number of multivariate representation schemes for the response function of a biochemical reaction system that can be used to analytically map the complex relationship between the biochemical factors and the system response. We then discuss how to use these schemes in order to approximate σ_j and η_j. Finally, we present details regarding the numerical implementation of the resulting approximation techniques.

1 Response function representation schemes

For ease of presentation, we will often base our discussion on a response function $R(\boldsymbol{w}) = R(w_1, w_2, w_3)$ that depends only on three factors of interest $\boldsymbol{w} = \{w_1, w_2, w_3\}$. Although extension to the case of J biochemical factors is straightforward, the required notation is cumbersome and makes key steps difficult to follow. For this reason, we use a trivariate response function to derive key equations and state the general form of these equations without proof.

1.1 TSMR

If the response function R is continuously differentiable in a neighborhood of $\boldsymbol{w} = 0$, then its Taylor series expansion about $\mathbf{0}$ is given by

$$\begin{aligned} R(w_1, w_2, w_3) = r_0 \; + \; & r_1(w_1) + r_2(w_2) + r_3(w_3) + r_{12}(w_1, w_2) \\ + \; & r_{13}(w_1, w_3) + r_{23}(w_2, w_3) + r_{123}(w_1, w_2, w_3), \end{aligned} \tag{S-1.1}$$

where

$$
\begin{aligned}
r_0 &:= R(\mathbf{0}) \\
r_1(w_1) &:= \sum_{m=1}^{\infty} \frac{1}{m!} \frac{\partial^m R(\mathbf{0})}{\partial w_1^m} \, w_1^m \\
r_2(w_2) &:= \sum_{m=1}^{\infty} \frac{1}{m!} \frac{\partial^m R(\mathbf{0})}{\partial w_2^m} \, w_2^m \\
r_3(w_3) &:= \sum_{m=1}^{\infty} \frac{1}{m!} \frac{\partial^m R(\mathbf{0})}{\partial w_3^m} \, w_3^m \\
r_{12}(w_1, w_2) &:= \sum_{m_1=1}^{\infty} \sum_{m_2=1}^{\infty} \frac{1}{m_1! \, m_2!} \frac{\partial^{m_1+m_2} R(\mathbf{0})}{\partial w_1^{m_1} \partial w_2^{m_2}} \, w_1^{m_1} w_2^{m_2} \\
r_{13}(w_1, w_3) &:= \sum_{m_1=1}^{\infty} \sum_{m_3=1}^{\infty} \frac{1}{m_1! \, m_3!} \frac{\partial^{m_1+m_3} R(\mathbf{0})}{\partial w_1^{m_1} \partial w_3^{m_3}} \, w_1^{m_1} w_3^{m_3} \\
r_{23}(w_2, w_3) &:= \sum_{m_2=1}^{\infty} \sum_{m_3=1}^{\infty} \frac{1}{m_2! \, m_3!} \frac{\partial^{m_2+m_3} R(\mathbf{0})}{\partial w_2^{m_2} \partial w_3^{m_3}} \, w_2^{m_2} w_3^{m_3} \\
r_{123}(w_1, w_2, w_3) &:= \sum_{m_1=1}^{\infty} \sum_{m_2=1}^{\infty} \sum_{m_3=1}^{\infty} \frac{1}{m_1! \, m_2! \, m_3!} \frac{\partial^{m_1+m_2+m_3} R(\mathbf{0})}{\partial w_1^{m_1} \partial w_2^{m_2} \partial w_3^{m_3}} \, w_1^{m_1} w_2^{m_2} w_3^{m_3}. \qquad \text{(S-1.2)}
\end{aligned}
$$

Clearly, the Taylor series expansion provides a representation of the system response R in terms of functions r, given by (S-1.2). We refer to the r's as *basis* functions. Note that r_0 is the value of R at the reference point $\mathbf{0}$. On the other hand, $r_1(w_1)$ summarizes the *singular* contribution of factor w_1 to the value of R, whereas, $r_{12}(w_1, w_2)$ summarizes the *joint* contribution of factors w_1 and w_2. Finally, $r_{123}(w_1, w_2, w_3)$ summarizes the joint contribution of all three factors to the value of R. Similar remarks apply for r_2, r_3, r_{13}, and r_{23}.

Although (S-1.2) provides analytical formulas for the basis functions, calculating these functions at a point $\boldsymbol{w}$ requires knowledge of the partial derivatives of R at the reference point $\mathbf{0}$, as well as evaluation of infinite sums, which is very difficult to do in practice. Note however that any basis function r given

by (S-1.2) is zero if one of its arguments equals zero. By using this property and (S-1.1), we have that

$$
\begin{aligned}
R(w_1, w_2, 0) &= r_0 + r_1(w_1) + r_2(w_2) + r_{12}(w_1, w_2) \\
R(w_1, 0, w_3) &= r_0 + r_1(w_1) + r_3(w_3) + r_{13}(w_1, w_3) \\
R(0, w_2, w_3) &= r_0 + r_2(w_2) + r_3(w_3) + r_{23}(w_2, w_3) \\
R(w_1, 0, 0) &= r_0 + r_1(w_1) \\
R(0, w_2, 0) &= r_0 + r_2(w_2) \\
R(0, 0, w_3) &= r_0 + r_3(w_3) \\
R(0, 0, 0) &= r_0,
\end{aligned}
$$

which results in

$$
\begin{aligned}
r_0 &= R(0,0,0) \\
r_1(w_1) &= R(w_1,0,0) - R(0,0,0) \\
r_2(w_2) &= R(0,w_2,0) - R(0,0,0) \\
r_3(w_3) &= R(0,0,w_3) - R(0,0,0) \\
r_{12}(w_1,w_2) &= R(w_1,w_2,0) - R(w_1,0,0) - R(0,w_2,0) + R(0,0,0) \\
r_{13}(w_1,w_3) &= R(w_1,0,w_3) - R(w_1,0,0) - R(0,0,w_3) + R(0,0,0) \\
r_{23}(w_2,w_3) &= R(0,w_2,w_3) - R(0,w_2,0) - R(0,0,w_3) + R(0,0,0) \\
r_{123}(w_1,w_2,w_3) &= R(w_1,w_2,w_3) - R(w_1,w_2,0) - R(w_1,0,w_3) - R(0,w_2,w_3) \\
&\quad + R(w_1,0,0) + R(0,w_2,0) + R(0,0,w_3) \\
&\quad - R(0,0,0). \qquad \text{(S-1.3)}
\end{aligned}
$$

These formulas provide a method for evaluating the basis functions r at some point $\boldsymbol{w}$. This can be done by calculating the system response at the corresponding w values suggested by the formulas. For example, evaluation of r_0 requires calculation of the system response at $w_1 = w_2 = w_3 = 0$, whereas, evaluation of $r_1(w_1)$ requires an additional calculation of the system response at $w_1, w_2 = w_3 = 0$. This can be done by solving the system of ordinary differential equations given by equations (1) and (2) in the Main text. We refer to the representation scheme given by (S-1.1) and (S-1.2) as *Taylor Series Model Representation* (TSMR).

1.2 FD-HDMR

We can extend the decomposition scheme given by (S-1.1) to the case of J biochemical factors and to functions that are not necessarily continuously differentiable. As a matter of fact, we can represent *any* response function R with J factors $\boldsymbol{w} = \{w_1, w_2, \ldots, w_J\}$ by

$$R(\boldsymbol{w}) = r_0 + \sum_{j=1}^{J} \sum_{1 \leq m_1 < \cdots < m_j \leq J} \cdots \sum r_{m_1 m_2 \cdots m_j}(w_{m_1}, w_{m_2}, \ldots, w_{m_j}). \tag{S-1.4}$$

The only requirement is that we must be able to *uniquely* determine the basis functions r from R. The representation of a multidimensional function R by (S-1.4) is known in the literature as *High-Dimensional Model Representation* (HDMR) [1,2].

A way to guarantee that we can uniquely determine r from the response function R is to consider basis functions that become zero if one of their arguments is zero. In this case, r can be determined by the classical Möbius inversion formula

$$r_{m_1 m_2 \cdots m_j}(w_{m_1}, w_{m_2}, \ldots, w_{m_j}) = \sum_{J \subseteq I} (-1)^{|I \setminus J|} R(\boldsymbol{w}_J), \tag{S-1.5}$$

which generalizes (S-1.3). In this formula, $I = \{m_1, m_2, \ldots, m_j\}$, $A \setminus B$ denotes the set difference between two sets A and B, $|A|$ denotes the number of elements in a set A (by convention, we set $|\emptyset| = 0$), and $\boldsymbol{w}_J$ is $\boldsymbol{w}$ with all variables, except the one indexed by J, set to zero.

Equations (S-1.4) and (S-1.5) express $R(\boldsymbol{w})$ as a superposition of system response values on lines, planes and hyperplanes passing through the reference point $\mathbf{0}$. For this reason, these equations lead to a system representation scheme known in the literature as cut-HDMR [1–3] or *Finite Difference* (FD) HDMR [4]. We adopt the second terminology here as being more appropriate for characterizing this type of HDMR. Clearly, the Taylor series expansion is a special case of FD-HDMR, with basis functions given by (S-1.2).

1.3 ANOVA-HDMR

Let us now assume that we can find invertible differentiable transformations g_j, which we can use to map the biochemical factors w_j into factors $u_j := g_j(w_j)$, that take values between 0 and 1. Let

$$P(u_1, u_2, u_3) := R(g_1^{-1}(u_1), g_2^{-1}(u_2), g_3^{-1}(u_3)). \tag{S-1.6}$$

The HDMR representation of P is given by

$$P(u_1, u_2, u_3) = p_0 + p_1(u_1) + p_2(u_2) + p_3(u_3) + p_{12}(u_1, u_2) + p_{13}(u_1, u_3) + p_{23}(u_2, u_3) + p_{123}(u_1, u_2, u_3). \quad \text{(S-1.7)}$$

If we consider basis functions p that integrate to zero over a *single* variable, then we can readily verify from (S-1.7) that

$$\begin{aligned}
p_0 &= \int_0^1 \int_0^1 \int_0^1 P(u_1, u_2, u_3) du_1 du_2 du_3 \\
p_1(u_1) &= \int_0^1 \int_0^1 P(u_1, u_2, u_3) du_2 du_3 - p_0 \\
p_2(u_2) &= \int_0^1 \int_0^1 P(u_1, u_2, u_3) du_1 du_3 - p_0 \\
p_3(u_3) &= \int_0^1 \int_0^1 P(u_1, u_2, u_3) du_1 du_2 - p_0 \\
p_{12}(u_1, u_2) &= \int_0^1 P(u_1, u_2, u_3) du_3 - p_1(u_1) - p_2(u_2) - p_0 \\
p_{13}(u_1, u_3) &= \int_0^1 P(u_1, u_2, u_3) du_2 - p_1(u_1) - p_3(u_3) - p_0 \\
p_{23}(u_2, u_3) &= \int_0^1 P(u_1, u_2, u_3) du_1 - p_2(u_2) - p_3(u_3) - p_0 \\
p_{123}(u_1, u_2, u_3) &= P(u_1, u_2, u_3) - p_{12}(u_1, u_2) - p_{13}(u_1, u_3) - p_{23}(u_2, u_3) \\
&\quad - p_1(u_1) - p_2(u_2) - p_3(u_3) - p_0. \quad \text{(S-1.8)}
\end{aligned}$$

Therefore, we can uniquely determine the basis functions p from P. By setting $u_j = g_j(w_j)$ in (S-1.7) and (S-1.8), and by employing (S-1.6), we obtain

$$R(w_1, w_2, w_3) = \rho_0 + \rho_1(w_1) + \rho_2(w_2) + \rho_3(w_3) + \rho_{12}(w_1, w_2) + \rho_{13}(w_1, w_3) + \rho_{23}(w_2, w_3) + \rho_{123}(w_1, w_2, w_3), \quad \text{(S-1.9)}$$

where

$$\rho_0 := p_0 = \int_{-\infty}^{\infty}\int_{-\infty}^{\infty}\int_{-\infty}^{\infty} R(w_1,w_2,w_3)g_1'(w_1)g_2'(w_2)g_3'(w_3)dw_1dw_2dw_3$$

$$\begin{aligned}
\rho_1(w_1) &:= p_1(g_1(w_1)) = \int_{-\infty}^{\infty}\int_{-\infty}^{\infty} R(w_1,w_2,w_3)g_2'(w_2)g_3'(w_3)dw_2dw_3 - \rho_0 \\
\rho_2(w_2) &:= p_2(g_2(w_2)) = \int_{-\infty}^{\infty}\int_{-\infty}^{\infty} R(w_1,w_2,w_3)g_1'(w_1)g_3'(w_3)dw_1dw_3 - \rho_0 \\
\rho_3(w_3) &:= p_3(g_3(w_3)) = \int_{-\infty}^{\infty}\int_{-\infty}^{\infty} R(w_1,w_2,w_3)g_1'(w_1)g_2'(w_2)dw_1dw_2 - \rho_0
\end{aligned}$$

$$\begin{aligned}
\rho_{12}(w_1,w_2) &:= p_{12}(g_1(w_1),g_2(w_2)) = \int_{-\infty}^{\infty} R(w_1,w_2,w_3)g_3'(w_3)dw_3 - \rho_1(w_1) - \rho_2(w_2) - \rho_0 \\
\rho_{13}(w_1,w_3) &:= p_{13}(g_1(w_1),g_3(w_3)) = \int_{-\infty}^{\infty} R(w_1,w_2,w_3)g_2'(w_2)dw_2 - \rho_1(w_1) - \rho_3(w_3) - \rho_0 \\
\rho_{23}(w_2,w_3) &:= p_{23}(g_2(w_2),g_3(w_3)) = \int_{-\infty}^{\infty} R(w_1,w_2,w_3)g_1'(w_1)dw_1 - \rho_2(w_2) - \rho_3(w_3) - \rho_0 \\
\rho_{123}(w_1,w_2,w_3) &:= p_{123}(g_1(w_1),g_2(w_2),g_3(w_3)) \\
&= R(w_1,w_2,w_3) - \rho_{12}(w_1,w_2) - \rho_{13}(w_1,w_3) - \rho_{23}(w_2,w_3) \\
&\quad -\rho_1(w_1) - \rho_2(w_2) - \rho_3(w_3) - \rho_0, \qquad \text{(S-1.10)}
\end{aligned}$$

with g' being the first-order derivative of g. For reasons to be explained in Section 2.3, the representation of a response function R by (S-1.9) and (S-1.10) is referred to in the literature as *Analysis-of-Variance* (ANOVA) HDMR [1–5]. Note that the basis functions ρ satisfy the following orthogonality conditions:

$$\begin{aligned}
&\int_{-\infty}^{\infty}\cdots\int_{-\infty}^{\infty} \rho_{j_1,\ldots,j_k}(w_{j_1},\ldots,w_{j_k})g_1'(w_1)\cdots g_J'(w_J)dw_1\cdots dw_J = 0, \\
&\int_{-\infty}^{\infty}\cdots\int_{-\infty}^{\infty} \rho_{j_1,\ldots,j_k}(w_{j_1},\ldots,w_{j_k})\rho_{j_1',\ldots,j_{k'}'}(w_{j_1'},\ldots,w_{j_{k'}'})g_1'(w_1)\cdots g_J'(w_J)dw_1\cdots dw_J = 0, \\
&\qquad (j_1,\ldots,j_k) \neq (j_1',\ldots,j_{k'}'), \qquad \text{(S-1.11)}
\end{aligned}$$

provided that the derivatives $g_j'(w_j)$ integrate to one.

2 Approximation of response variances

In this section, we assume that the biochemical factors of interest are statistically independent random variables $W_1, W_2, \ldots, W_J$ that follow zero-mean Gaussian distributions with standard deviations

$\lambda_1, \lambda_2, \ldots, \lambda_J$, respectively. In this case, the response $R(\boldsymbol{W})$ of the biochemical reaction system, where $\boldsymbol{W} = \{W_1, W_2, \ldots, W_J\}$, will be a random variable as well. We are interested in evaluating the following response variances:

$$\begin{aligned} V_j &:= \text{Var}[\text{E}[R(\boldsymbol{W}) \mid W_j]] \\ V_{jj'} &:= \text{Var}[\text{E}[R(\boldsymbol{W}) \mid W_j, W_{j'}]] - V_j - V_{j'}. \end{aligned} \tag{S-1.12}$$

We can then calculate the (second-order) SESI's and JESI's by means of

$$\sigma_j = \frac{V_j}{V} \qquad \text{and} \qquad \eta_j = \frac{U_j}{V}\,, \tag{S-1.13}$$

where

$$U_j := \sum_{j'=1, j' \neq j}^{J} V_{jj'} \qquad \text{and} \qquad V := \sum_{j=1}^{J} V_j + \sum_{j=1}^{J-1} \sum_{j'=j+1}^{J} V_{jj'}. \tag{S-1.14}$$

In most applications of interest however it is very difficult to evaluate the previous variances due to the complexity of the response function R. We can address this problem by replacing the response function with a simpler function $\widehat{R}(w_1, w_2, \ldots, w_J)$ that will allow us to approximate the response variances given by (S-1.12). In the following, we discuss various approximations obtained by employing the previously discussed representation schemes.

2.1 TSMR

As we mentioned in Section 1.1, the two main problems associated with the basis functions of TSMR, given by (S-1.2), is the need to calculate high-order partial derivatives of the response function and evaluate infinite sums. To address these problems, we can approximate the basis functions by assuming that the response function is sufficiently smooth in a neighborhood around $\mathbf{0}$ so that partial derivatives of order greater than two are negligible. In this case, we can approximate the response function $R(w_1, w_2, w_3)$ by

$$\widehat{R}(w_1, w_2, w_3) = \widehat{r}_0 + \widehat{r}_1(w_1) + \widehat{r}_2(w_2) + \widehat{r}_3(w_3) + \widehat{r}_{12}(w_1, w_2) + \widehat{r}_{13}(w_1, w_3) + \widehat{r}_{23}(w_2, w_3),$$

where

$$\begin{aligned} \widehat{r}_0 &:= R(\mathbf{0}) \\ \widehat{r}_1(w_1) &:= \frac{\partial R(\mathbf{0})}{\partial w_1}\, w_1 + \frac{1}{2}\frac{\partial^2 R(\mathbf{0})}{\partial w_1^2}\, w_1^2 \\ \widehat{r}_2(w_2) &:= \frac{\partial R(\mathbf{0})}{\partial w_2}\, w_2 + \frac{1}{2}\frac{\partial^2 R(\mathbf{0})}{\partial w_2^2}\, w_2^2 \end{aligned}$$

$$
\begin{aligned}
\widehat{r}_3(w_3) &:= \frac{\partial R(\mathbf{0})}{\partial w_3}\, w_3 + \frac{1}{2}\frac{\partial^2 R(\mathbf{0})}{\partial w_3^2}\, w_3^2 \\
\widehat{r}_{12}(w_1, w_2) &:= \frac{\partial^2 R(\mathbf{0})}{\partial w_1 \partial w_2}\, w_1 w_2 \\
\widehat{r}_{13}(w_1, w_3) &:= \frac{\partial^2 R(\mathbf{0})}{\partial w_1 \partial w_3}\, w_1 w_3 \\
\widehat{r}_{23}(w_2, w_3) &:= \frac{\partial^2 R(\mathbf{0})}{\partial w_2 \partial w_3}\, w_2 w_3 \,,
\end{aligned}
$$

since $r_{123}(w_1, w_2, w_3) = 0$ in this case. By employing the statistical independence of W_1, W_2, and W_3, we can show that the variances associated with the approximate response function $\widehat{R}(w_1, w_2, w_3)$ satisfy:

$$
\begin{aligned}
\widehat{V} &= d_1^2\lambda_1^2 + d_2^2\lambda_2^2 + d_3^2\lambda_3^2 + \frac{1}{2}\, d_{11}^2\lambda_1^4 + \frac{1}{2}\, d_{22}^2\lambda_2^4 + \frac{1}{2}\, d_{33}^2\lambda_3^4 + d_{12}^2\lambda_1^2\lambda_2^2 + d_{13}^2\lambda_1^2\lambda_3^2 + d_{23}^2\lambda_2^2\lambda_3^2 \\
\widehat{V}_1 &= d_1^2\lambda_1^2 + \frac{1}{2}\, d_{11}^2\lambda_1^4 \\
\widehat{V}_2 &= d_2^2\lambda_2^2 + \frac{1}{2}\, d_{22}^2\lambda_2^4 \\
\widehat{V}_3 &= d_3^2\lambda_3^2 + \frac{1}{2}\, d_{33}^2\lambda_3^4 \\
\widehat{V}_{12} &= d_{12}^2\lambda_1^2\lambda_2^2 \\
\widehat{V}_{13} &= d_{13}^2\lambda_1^2\lambda_3^2 \\
\widehat{V}_{23} &= d_{23}^2\lambda_2^2\lambda_3^2 \,,
\end{aligned}
\qquad \text{(S-1.15)}
$$

where d_j is the first-order partial derivative of R with respect to w_j at $\mathbf{0}$ and $d_{jj'}$ is the second-order partial derivative of R with respect to w_j and $w_{j'}$ at $\mathbf{0}$. To show (S-1.15), we have used the fact that W_j follows a Gaussian distribution with zero mean and standard deviation λ_j, which implies $\mathrm{E}[W_j^3] = 0$, $\mathrm{E}[W_j^4] = 3\lambda_j^4$. As a consequence of (S-1.13), (S-1.14), and (S-1.15), we obtain the following approximations to the SESI's and JESI's (expressed for the general case of J biochemical factors):

$$
\boxed{
\begin{aligned}
&\widehat{\sigma}_j = \frac{\widehat{V}_j}{\widehat{V}}, \qquad \widehat{\eta}_j = \frac{\widehat{U}_j}{\widehat{V}} \\
&\widehat{V}_j = \lambda_j^2 d_j^2 + \frac{1}{2}\, \lambda_j^4 d_{jj}^2 \\
&\widehat{V}_{jj'} = \lambda_j^2 \lambda_{j'}^2 d_{jj'}^2 \\
&\widehat{U}_j = \sum_{j'=1, j'\neq j}^{J} \widehat{V}_{jj'} \\
&\widehat{V} = \sum_{j=1}^{J} \widehat{V}_j + \sum_{j=1}^{J-1} \sum_{j'=j+1}^{J} \widehat{V}_{jj'}
\end{aligned}
}
\qquad \text{(S-1.16)}
$$

We respectively refer to $\widehat{\sigma}_j$ and $\widehat{\eta}_j$, given by (S-1.16), as the SESI's and JESI's obtained by *Derivative Approximation* (DA).

2.2 FD-HDMR

2.2.1 Polynomial approximation

We may obtain a better approximation of the sensitivity indices σ_j and η_j by assuming that the response function is sufficiently smooth in a neighborhood around $\mathbf{0}$ so that partial derivatives of order greater than two with respect to one variable and partial derivatives that involve more than two variables are negligible. In this case, we can approximate the response function $R(w_1, w_2, w_3)$ by

$$\widehat{R}(w_1, w_2, w_3) = \widehat{r}_0 + \widehat{r}_1(w_1) + \widehat{r}_2(w_2) + \widehat{r}_3(w_3) + \widehat{r}_{12}(w_1, w_2) + \widehat{r}_{13}(w_1, w_3) + \widehat{r}_{23}(w_2, w_3), \quad \text{(S-1.17)}$$

where

$$\begin{aligned}
\widehat{r}_0 &:= R(\mathbf{0}) \\
\widehat{r}_1(w_1) &:= \frac{\partial R(\mathbf{0})}{\partial w_1}\, w_1 + \frac{1}{2}\frac{\partial^2 R(\mathbf{0})}{\partial w_1^2}\, w_1^2 \\
\widehat{r}_2(w_2) &:= \frac{\partial R(\mathbf{0})}{\partial w_2}\, w_2 + \frac{1}{2}\frac{\partial^2 R(\mathbf{0})}{\partial w_2^2}\, w_2^2 \\
\widehat{r}_3(w_3) &:= \frac{\partial R(\mathbf{0})}{\partial w_3}\, w_3 + \frac{1}{2}\frac{\partial^2 R(\mathbf{0})}{\partial w_3^2}\, w_3^2 \\
\widehat{r}_{12}(w_1, w_2) &:= \frac{\partial^2 R(\mathbf{0})}{\partial w_1 \partial w_2}\, w_1 w_2 + \frac{1}{2}\frac{\partial^3 R(\mathbf{0})}{\partial w_1^2 \partial w_2}\, w_1^2 w_2 + \frac{1}{2}\frac{\partial^3 R(\mathbf{0})}{\partial w_1 \partial w_2^2}\, w_1 w_2^2 + \frac{1}{4}\frac{\partial^4 R(\mathbf{0})}{\partial w_1^2 \partial w_2^2}\, w_1^2 w_2^2 \\
\widehat{r}_{13}(w_1, w_3) &:= \frac{\partial^2 R(\mathbf{0})}{\partial w_1 \partial w_3}\, w_1 w_3 + \frac{1}{2}\frac{\partial^3 R(\mathbf{0})}{\partial w_1^2 \partial w_3}\, w_1^2 w_3 + \frac{1}{2}\frac{\partial^3 R(\mathbf{0})}{\partial w_1 \partial w_3^2}\, w_1 w_3^2 + \frac{1}{4}\frac{\partial^4 R(\mathbf{0})}{\partial w_1^2 \partial w_3^2}\, w_1^2 w_3^2 \\
\widehat{r}_{23}(w_2, w_3) &:= \frac{\partial^2 R(\mathbf{0})}{\partial w_2 \partial w_3}\, w_2 w_3 + \frac{1}{2}\frac{\partial^3 R(\mathbf{0})}{\partial w_2^2 \partial w_3}\, w_2^2 w_3 + \frac{1}{2}\frac{\partial^3 R(\mathbf{0})}{\partial w_2 \partial w_3^2}\, w_2 w_3^2 + \frac{1}{4}\frac{\partial^4 R(\mathbf{0})}{\partial w_2^2 \partial w_3^2}\, w_2^2 w_3^2. \quad \text{(S-1.18)}
\end{aligned}$$

Due to difficulties in numerically evaluating high-order derivatives with sufficient accuracy, we may not be able to use (S-1.18) to derive sufficiently good DA approximations of the sensitivity indices. However, this equation motivates us to set

$$\begin{aligned}
\widehat{r}_j(w_j) &= \alpha_{j,1} w_j + \alpha_{j,2}\, w_j^2 \\
\widehat{r}_{jj'}(w_j, w_{j'}) &= \alpha_{jj',1}\, w_j w_{j'} + \alpha_{jj',2}\, w_j^2 w_{j'} + \alpha_{jj',3}\, w_j w_{j'}^2 + \alpha_{jj',4}\, w_j^2 w_{j'}^2, \quad \text{(S-1.19)}
\end{aligned}$$

where the α's are parameters whose values must be appropriately determined so that $\widehat{R}$, given by (S-1.17) and (S-1.19), sufficiently approximates the response function R. We will be discussing a practical method to address this problem in Section 3 of this supplement.

Clearly, the previous approach is based on approximating the first- and second-order basis functions associated with the FD-HDMR given by (S-1.4) with the polynomials given by (S-1.19). If $\widehat{R}(\boldsymbol{w})$ is sufficiently close to $R(\boldsymbol{w})$ in a neighborhood around $\mathbf{0}$, then the parameters α will coincide with the partial derivatives of R associated with (S-1.18). Note that the approximating basis functions $\widehat{r}$ given by (S-1.19) satisfy the necessary condition of becoming zero if one of their arguments equals zero.

As a consequence of (S-1.13) and (S-1.14), by employing the statistical independence of the W_j's, and by using the fact that W_j follows a Gaussian distribution with zero mean and standard deviation λ_j, in which case $\mathrm{E}[W_j^3] = 0$ and $\mathrm{E}[W_j^4] = 3\lambda_j^4$, we obtain the following approximations to the SESI's and JESI's:

$$\boxed{\begin{aligned}
&\widehat{\sigma}_j = \frac{\widehat{V}_j}{\widehat{V}}, \qquad \widehat{\eta}_j = \frac{\widehat{U}_j}{\widehat{V}} \\
&\widehat{V}_j = \lambda_j^2\alpha_{j,1}^2 + 2\lambda_j^4\alpha_{j,2}^2 + 2\lambda_j^2\alpha_{j,1}\left(\sum_{m=1}^{j-1}\lambda_m^2\alpha_{mj,2} + \sum_{m=j+1}^{J}\lambda_m^2\alpha_{jm,3}\right) \\
&\qquad + \lambda_j^2\left(\sum_{m=1}^{j-1}\lambda_m^2\alpha_{mj,2} + \sum_{m=j+1}^{J}\lambda_m^2\alpha_{jm,3}\right)^2 \\
&\qquad + 4\lambda_j^4\alpha_{j,2}\left(\sum_{m=1}^{j-1}\lambda_m^2\alpha_{mj,4} + \sum_{m=j+1}^{J}\lambda_m^2\alpha_{jm,4}\right) \\
&\qquad + 2\lambda_j^4\left(\sum_{m=1}^{j-1}\lambda_m^2\alpha_{mj,4} + \sum_{m=j+1}^{J}\lambda_m^2\alpha_{jm,4}\right)^2 \\
&\widehat{V}_{jj'} = \lambda_j^2\lambda_{j'}^2\alpha_{jj',1}^2 + 2\lambda_j^4\lambda_{j'}^2\alpha_{jj',2}^2 + 2\lambda_j^2\lambda_{j'}^4\alpha_{jj',3}^2 + 4\lambda_j^4\lambda_{j'}^4\alpha_{jj',4}^2 \\
&\widehat{U}_j = \sum_{j'=1, j'\neq j}^{J}\widehat{V}_{jj'} \\
&\widehat{V} = \sum_{j=1}^{J}\widehat{V}_j + \sum_{j=1}^{J-1}\sum_{j'=j+1}^{J}\widehat{V}_{jj'}
\end{aligned}} \qquad \text{(S-1.20)}$$

Note that (S-1.20) is a special case of equations 35 and 36 in [6]. We respectively refer to $\widehat{\sigma}_j$ and $\widehat{\eta}_j$, given by (S-1.20), as the SESI's and JESI's obtained by *P̲olynomial A̲pproximation* (PA) of the FD-HDMR.

2.2.2 Gauss-Hermite integration

We can derive another approximation of the sensitivity indices by assuming that the partial derivatives of the response function in a neighborhood of $\mathbf{0}$ that involve more than two factors are negligible. In this case, we can approximate the response function $R(w_1, w_2, w_3)$ by

$$\widehat{R}(w_1, w_2, w_3) = r_0 + r_1(w_1) + r_2(w_2) + r_3(w_3) + r_{12}(w_1, w_2) + r_{13}(w_1, w_3) + r_{23}(w_2, w_3), \quad \text{(S-1.21)}$$

where

$$\begin{aligned}
r_0 &= R(\mathbf{0}) \\
r_1(w_1) &= \sum_{m=1}^{\infty} \frac{1}{m!} \frac{\partial^m R(\mathbf{0})}{\partial w_1^m} \, w_1^m \\
r_2(w_2) &= \sum_{m=1}^{\infty} \frac{1}{m!} \frac{\partial^m R(\mathbf{0})}{\partial w_2^m} \, w_2^m \\
r_3(w_3) &= \sum_{m=1}^{\infty} \frac{1}{m!} \frac{\partial^m R(\mathbf{0})}{\partial w_3^m} \, w_3^m \\
r_{12}(w_1, w_2) &= \sum_{m_1=1}^{\infty} \sum_{m_2=1}^{\infty} \frac{1}{m_1! m_2!} \frac{\partial^{m_1+m_2} R(\mathbf{0})}{\partial w_1^{m_1} \partial w_2^{m_2}} \, w_1^{m_1} w_2^{m_2} \\
r_{13}(w_1, w_3) &= \sum_{m_1=1}^{\infty} \sum_{m_3=1}^{\infty} \frac{1}{m_1! m_3!} \frac{\partial^{m_1+m_3} R(\mathbf{0})}{\partial w_1^{m_1} \partial w_3^{m_3}} \, w_1^{m_1} w_3^{m_3} \\
r_{23}(w_2, w_3) &= \sum_{m_2=1}^{\infty} \sum_{m_3=1}^{\infty} \frac{1}{m_2! m_3!} \frac{\partial^{m_2+m_3} R(\mathbf{0})}{\partial w_2^{m_2} \partial w_3^{m_3}} \, w_2^{m_2} w_3^{m_3},
\end{aligned} \quad \text{(S-1.22)}$$

since $r_{123}(w_1, w_2, w_3) = 0$ in this case. We expect that this approximation will be more accurate than the one considered in (S-1.17) and (S-1.18), since the first- and second-order basis functions are exactly the same as the corresponding basis functions given by (S-1.2). Note that we can obtain the approximation given by (S-1.21) by simply truncating the third- and higher-order terms in the FD-HDMR of the response function R, given by (S-1.4), without making any reference to the derivatives of R.

Since the basis functions r given by(S-1.22) become zero if one of their arguments is zero, we can relate them to the system response R by means of (S-1.3). As a consequence of (S-1.3) and (S-1.21), we obtain

the following decomposition for $\widehat{R}$ (expressed for the general case of J biochemical factors):

$$\widehat{R}(\boldsymbol{w}) = \psi_0 - (J-2)\sum_{j=1}^{J}\psi_j(w_j) + \sum_{j=1}^{J-1}\sum_{j'=j+1}^{J}\psi_{jj'}(w_j, w_{j'}), \tag{S-1.23}$$

where

$$\begin{aligned}
\psi_0 &:= \frac{(J-1)(J-2)}{2}\, R(0,0,\ldots,0) \\
\psi_j(w_j) &:= R(0,\ldots,0,w_j,0,\ldots,0) \\
\psi_{jj'}(w_j, w_{j'}) &:= R(0,\ldots,0,w_j,0,\ldots,0,w_{j'},0,\ldots,0)\,.
\end{aligned}$$

By taking conditional and unconditional expectations on both sides of (S-1.23), and by using the statistical independence of the biochemical factors, we obtain

$$\begin{aligned}
e_0 &:= \mathrm{E}[\widehat{R}(\boldsymbol{W})] \\
&= \psi_0 - (J-2)\sum_{m=1}^{J}\mathrm{E}[\psi_m(W_m)] + \sum_{m=1}^{J-1}\sum_{m'=m+1}^{J}\mathrm{E}[\psi_{mm'}(W_m, W_{m'})] \\
e_j(w_j) &:= \mathrm{E}[\widehat{R}(\boldsymbol{W}) \mid W_j = w_j] \\
&= \psi_0 - (J-2)\sum_{m=1}^{J}\mathrm{E}[\psi_m(W_m) \mid W_j = w_j] \\
&\quad + \sum_{m=1}^{J-1}\sum_{m'=m+1}^{J}\mathrm{E}[\psi_{mm'}(W_m, W_{m'}) \mid W_j = w_j] \\
e_{jj'}(w_j, w_{j'}) &:= \mathrm{E}[R(\boldsymbol{W}) \mid W_j = w_j, W_{j'} = w_{j'}] \\
&= \psi_0 - (J-2)\sum_{m=1}^{J}\mathrm{E}[\psi_m(W_m) \mid W_j = w_j, W_{j'} = w_{j'}] \\
&\quad + \sum_{m=1}^{J-1}\sum_{m'=m+1}^{J}\mathrm{E}[\psi_{mm'}(W_m, W_{m'}) \mid W_j = w_j, W_{j'} = w_{j'}],
\end{aligned} \tag{S-1.24}$$

where

$$\mathrm{E}[\psi_m(W_m) \mid W_j = w_j] \;=\; \begin{cases} \psi_j(w_j), & \text{if } m = j \\ \mathrm{E}[\psi_m(W_m)], & \text{otherwise} \end{cases}$$

$$\mathrm{E}[\psi_m(W_m) \mid W_j = w_j, W_{j'} = w_{j'}] \;=\; \begin{cases} \psi_j(w_j), & \text{if } m = j \\ \psi_{j'}(w_{j'}), & \text{if } m = j' \\ \mathrm{E}[\psi_m(W_m)], & \text{otherwise} \end{cases}, \quad \text{for } j < j'$$

$$\mathrm{E}[\psi_{mm'}(W_m, W_{m'}) \mid W_j = w_j] \;=\; \begin{cases} \mathrm{E}[\psi_{mj}(W_m, w_j)], & \text{if } m < j, m' = j \\ \mathrm{E}[\psi_{jm'}(w_j, W_{m'})], & \text{if } m = j, m' > j \\ \mathrm{E}[\psi_{mm'}(W_m, W_{m'})], & \text{otherwise} \end{cases}$$

$$\mathrm{E}[\psi_{mm'}(W_m, W_{m'}) \mid W_j = w_j, W_{j'} = w_{j'}] \;=\; \begin{cases} \psi_{jj'}(w_j, w_{j'}), & \text{if } m = j, m' = j' \\ \mathrm{E}[\psi_{mj}(W_m, w_j)], & \text{if } m < j, m' = j \\ \mathrm{E}[\psi_{mj'}(W_m, w_{j'})], & \text{if } m \neq j, m' = j' \\ \mathrm{E}[\psi_{jm'}(w_j, W_{m'})], & \text{if } m = j, m' \neq j' \\ \mathrm{E}[\psi_{j'm'}(w_{j'}, W_{m'})], & \text{if } m = j', m' > j' \\ \mathrm{E}[\psi_{mm'}(W_m, W_{m'})], & \text{otherwise} \end{cases}, \quad \text{for } j < j'. \tag{S-1.25}$$

Finally, to compute the conditional variances of the response function $\widehat{R}$, note that

$$\mathrm{Var}[\mathrm{E}[\widehat{R}(\boldsymbol{W}) \mid W_j]] \;=\; \mathrm{Var}[e_j(W_j)] = \mathrm{E}[e_j^2(W_j)] - e_0^2$$

$$\mathrm{Var}[\mathrm{E}[\widehat{R}(\boldsymbol{W}) \mid W_j, W_{j'}]] \;=\; \mathrm{Var}[e_{jj'}(W_j, W_{j'})] = \mathrm{E}[e_{jj'}^2(W_j, W_{j'})] - e_0^2, \tag{S-1.26}$$

since

$$\mathrm{E}[e_j(W_j)] \;=\; \mathrm{E}[\mathrm{E}[\widehat{R}(\boldsymbol{W}) \mid W_j]] = \mathrm{E}[\widehat{R}(\boldsymbol{W})]$$

$$\mathrm{E}[e_{jj'}(W_j, W_{j'})] \;=\; \mathrm{E}[\mathrm{E}[\widehat{R}(\boldsymbol{W}) \mid W_j, W_{j'}]] = \mathrm{E}[\widehat{R}(\boldsymbol{W})],$$

by virtue of the fact that $\mathrm{E}[\mathrm{E}[Y|X]] = \mathrm{E}[Y]$.

As a consequence of (S-1.13), (S-1.14), and (S-1.26), we now obtain the following approximations to the SESI's and JESI's:

$$\begin{array}{|c|}\hline \widehat{\sigma}_j = \frac{\widehat{V}_j}{\widehat{V}}, \qquad \widehat{\eta}_j = \frac{\widehat{U}_j}{\widehat{V}} \\ \widehat{V}_j = \mathrm{E}[e_j^2(W_j)] - e_0^2 \\ \widehat{V}_{jj'} = \mathrm{E}[e_{jj'}^2(W_j, W_{j'})] - \widehat{V}_j - \widehat{V}_{j'} - e_0^2 \\ \widehat{U}_j = \sum_{j'=1, j' \neq j}^{J} \widehat{V}_{jj'} \\ \widehat{V} = \sum_{j=1}^{J} \widehat{V}_j + \sum_{j=1}^{J-1} \sum_{j'=j+1}^{J} \widehat{V}_{jj'} \\ \hline \end{array} \qquad \text{(S-1.27)}$$

with e_0, e_j, and $e_{jj'}$ given by (S-1.24). Note that evaluation of the expectations of these quantities requires only one- and two-dimensional integrations. This can be done by a standard Gauss-Hermite integration procedure, as we explain in Section 3. We respectively refer to $\widehat{\sigma}_j$ and $\widehat{\eta}_j$, given by Equation S-1.27, as the SESI's and JESI's obtained by *Gauss-Hermite Integration* (GHI) of the FD-HDMR.

2.3 ANOVA-HDMR

Equation (S-1.7) and the fact that the basis functions p integrate to zero over a single variable imply

$$\begin{aligned} \int_0^1 \int_0^1 \int_0^1 P^2(u_1, u_2, u_3) du_1 du_2 du_3 = p_0^2 &+ \int_0^1 p_1^2(u_1) du_1 + \int_0^1 p_2^2(u_2) du_2 + \int_0^1 p_3^2(u_3) du_3 \\ &+ \int_0^1 \int_0^1 p_{12}^2(u_1, u_2) du_1 du_2 \\ &+ \int_0^1 \int_0^1 p_{13}^2(u_1, u_3) du_1 du_3 \\ &+ \int_0^1 \int_0^1 p_{23}^2(u_2, u_3) du_2 du_3 \\ &+ \int_0^1 \int_0^1 \int_0^1 p_{123}^2(u_1, u_2, u_3) du_1 du_2 du_3 \, . \end{aligned} \qquad \text{(S-1.28)}$$

If we assume that the biochemical factors of interest are statistically independent random variables W_1, W_2, and W_3, with cumulative distribution functions $g_1(w_1)$, $g_2(w_2)$, and $g_3(w_3)$, respectively, then (S-1.28), together with (S-1.6), (S-1.8), and (S-1.10), implies that

$$V = V_1 + V_2 + V_3 + V_{12} + V_{13} + V_{23} + V_{123}, \qquad \text{(S-1.29)}$$

where

$$
\begin{aligned}
V &:= \text{Var}[R(W_1, W_2, W_3)] \\
V_1 &:= \text{Var}[\text{E}[R(W_1, W_2, W_3) \mid W_1]] = \int_{-\infty}^{\infty} \rho_1^2(w_1) g_1'(w_1) dw_1 \\
V_2 &:= \text{Var}[\text{E}[R(W_1, W_2, W_3) \mid W_2]] = \int_{-\infty}^{\infty} \rho_2^2(w_2) g_2'(w_2) dw_2 \\
V_3 &:= \text{Var}[\text{E}[R(W_1, W_2, W_3) \mid W_3]] = \int_{-\infty}^{\infty} \rho_3^2(w_3) g_3'(w_3) dw_3 \\
V_{12} &:= \text{Var}[\text{E}[R(W_1, W_2, W_3) \mid W_1, W_2]] - V_1 - V_2 = \int_{-\infty}^{\infty} \int_{-\infty}^{\infty} \rho_{12}^2(w_1, w_2) g_1'(w_1) g_2'(w_2) dw_1 dw_2 \geq 0 \\
V_{13} &:= \text{Var}[\text{E}[R(W_1, W_2, W_3) \mid W_1, W_3]] - V_1 - V_3 = \int_{-\infty}^{\infty} \int_{-\infty}^{\infty} \rho_{13}^2(w_1, w_3) g_1'(w_1) g_3'(w_3) dw_1 dw_3 \geq 0 \\
V_{23} &:= \text{Var}[\text{E}[R(W_1, W_2, W_3) \mid W_2, W_3]] - V_2 - V_3 = \int_{-\infty}^{\infty} \int_{-\infty}^{\infty} \rho_{23}^2(w_2, w_3) g_2'(w_2) g_3'(w_3) dw_2 dw_3 \geq 0 \\
V_{123} &:= V - V_{12} - V_{13} - V_{23} - V_1 - V_2 - V_3 \\
&= \int_{-\infty}^{\infty} \int_{-\infty}^{\infty} \int_{-\infty}^{\infty} \rho_{123}^2(w_1, w_2, w_3) g_1'(w_1) g_2'(w_2) g_3'(w_3) dw_1 dw_2 dw_3 \geq 0, \qquad \text{(S-1.30)}
\end{aligned}
$$

since $g_1'(w_1)$, $g_2'(w_2)$, and $g_3'(w_3)$ are the probability density functions of W_1, W_2, and W_3, respectively. The variance decomposition scheme given by Equations 6 and 7 in the Main text is a general version of the decomposition given by (S-1.29) and (S-1.30) for the case of J biochemical factors. This decomposition is closely related to *analysis of variance* (ANOVA) techniques in statistics [5, 7, 8]. For this reason, the representation of the response function R by (S-1.9) and (S-1.10) is referred to in the literature as ANOVA-HDMR.

Note that (S-1.29) can be shown in a trivial manner by adding all V's in (S-1.30). However, by using (S-1.6), (S-1.8), (S-1.10), and (S-1.28), we can show that, when W_1, W_2, and W_3 are statistically independent, then $V_{12}, V_{13}, V_{23}, V_{123} \geq 0$, which is a crucial property for appropriately defining the variance-based sensitivity indices we consider in this paper. Moreover, we can show that these quantities can be directly evaluated from the basis functions of the ANOVA-HDMR of the response function $R(\boldsymbol{w})$ by means of (S-1.30). As a consequence, we can use ANOVA-HDMR to develop an efficient approximation technique for the sensitivity indices σ_j and η_j. We can do this by sufficiently approximating the response $R(w_1, w_2, w_3)$ by a function

$$
\widehat{R}(w_1, w_2, w_3) = \widehat{\rho}_0 + \widehat{\rho}_1(w_1) + \widehat{\rho}_2(w_2) + \widehat{\rho}_3(w_3) + \widehat{\rho}_{12}(w_1, w_2) + \widehat{\rho}_{13}(w_1, w_3) + \widehat{\rho}_{23}(w_2, w_3), \qquad \text{(S-1.31)}
$$

where the approximating basis functions $\widehat{\rho}$ must be appropriately chosen so that they satisfy the necessary

orthogonality conditions, given by (S-1.11), and allow efficient evaluation of the integrals in (S-1.30).

There are several potential choices for the approximating basis functions $\widehat{\rho}$, such as polynomials, exponentials, splines, etc. However, for the case of statistically independent zero-mean Gaussian biochemical factors, the simplest choice is based on the following first- and second-order Hermite polynomials:

$$H_1(x) = x \qquad \text{and} \qquad H_2(x) = \frac{x^2 - 1}{\sqrt{2}}\,.$$

Note that these polynomials are orthonormal over the standard Gaussian distribution, satisfying

$$\begin{aligned}
&\int_{-\infty}^{\infty} H_1(x)\,\frac{1}{\sqrt{2\pi}}\,e^{-x^2/2}dx = \int_{-\infty}^{\infty} H_2(x)\,\frac{1}{\sqrt{2\pi}}\,e^{-x^2/2}dx = 0\\
&\int_{-\infty}^{\infty} H_1^2(x)\,\frac{1}{\sqrt{2\pi}}\,e^{-x^2/2}dx = \int_{-\infty}^{\infty} H_2^2(x)\,\frac{1}{\sqrt{2\pi}}\,e^{-x^2/2}dx = 1\\
&\int_{-\infty}^{\infty} H_1(x)H_2(x)\,\frac{1}{\sqrt{2\pi}}\,e^{-x^2/2}dx = 0. \qquad \text{(S-1.32)}
\end{aligned}$$

In this case, we set

$$\begin{aligned}
\widehat{\rho}_j(w_j) &= \alpha_{j,1}\frac{w_j}{\lambda_j} + \frac{\alpha_{j,2}}{\sqrt{2}}\left(\frac{w_j^2}{\lambda_j^2} - 1\right)\\
\widehat{\rho}_{jj'}(w_j, w_{j'}) &= \alpha_{jj',1}\frac{w_j w_{j'}}{\lambda_j\lambda_{j'}} + \frac{\alpha_{jj',2}}{\sqrt{2}}\left(\frac{w_j^2}{\lambda_j^2} - 1\right)\frac{w_{j'}}{\lambda_{j'}} + \frac{\alpha_{jj',3}}{\sqrt{2}}\frac{w_j}{\lambda_j}\left(\frac{w_{j'}^2}{\lambda_{j'}^2} - 1\right)\\
&\quad + \frac{\alpha_{jj',4}}{2}\left(\frac{w_j^2}{\lambda_j^2} - 1\right)\left(\frac{w_{j'}^2}{\lambda_{j'}^2} - 1\right). \qquad \text{(S-1.33)}
\end{aligned}$$

Note that, since the biochemical factors W_j are statistically independent zero-mean Gaussian random variables with standard deviations given by λ_j, these approximations satisfy the necessary orthogonality conditions given by (S-1.11).

By using (S-1.30) and the orthonormality of the Hermite polynomials H_1 and H_2, given by (S-1.32), we can obtain the following approximations to the SESI's and JESI's (expressed for the general case of J biochemical factors):

$$
\boxed{\begin{gathered}
\widehat{\sigma}_j = \frac{\widehat{V}_j}{\widehat{V}}, \qquad \widehat{\eta}_j = \frac{\widehat{U}_j}{\widehat{V}} \\
\widehat{V}_j = \alpha_{j,1}^2 + \alpha_{j,2}^2 \\
\widehat{V}_{jj'} = \alpha_{jj',1}^2 + \alpha_{jj',2}^2 + \alpha_{jj',3}^2 + \alpha_{jj',4}^2 \\
\widehat{U}_j = \sum_{j'=1, j' \neq j}^{J} \widehat{V}_{jj'} \\
\widehat{V} = \sum_{j=1}^{J} \widehat{V}_j + \sum_{j=1}^{J-1} \sum_{j'=j+1}^{J} \widehat{V}_{jj'}
\end{gathered}} \tag{S-1.34}
$$

We respectively refer to $\widehat{\sigma}_j$ and $\widehat{\eta}_j$, given by (S-1.34), as the SESI's and JESI's obtained by *Orthonormal Hermite Approximation* (OHA) of the ANOVA-HDMR.

3 Numerical implementation

We now discuss the numerical implementation of the approximation techniques we presented in the previous section. Some techniques can be implemented in a straightforward manner, while others require more involved implementation steps.

3.1 TSMR

Approximation of the SESI's and JESI's by means of (S-1.16) requires evaluation of the first- and second-order partial derivatives of the response function $R(\boldsymbol{w})$ at $\boldsymbol{w} = \mathbf{0}$, given by

$$
d_j = \frac{\partial R(\mathbf{0})}{\partial w_j} \qquad \text{and} \qquad d_{jj'} = \frac{\partial^2 R(\mathbf{0})}{\partial w_j \partial w_{j'}} \, .
$$

Unfortunately, accurate evaluation of these derivatives is not an easy task [9]. We may express them in terms of concentration sensitivities and analytically derive a system of differential equations that govern the dynamic evolution of such sensitivities. Then, evaluation of the response derivatives will require simultaneous integration of the sensitivity equations together with the differential equations governing the underlying molecular concentration dynamics. Most often, this step cannot be implemented in a reasonable time due to stiffness of the resulting differential equations [10]. As a consequence, the derivatives are usually approximated by finite-differences. However, the resulting approximations must be carefully used, since it is difficult to theoretically predict, control, and numerically evaluate the accuracy of finite-difference approximations of derivatives [10].

In this work, we use symmetric finite-difference approximations of the derivatives. A symmetric finite-difference approximation of the first-order partial derivative d_j of $R(\boldsymbol{w})$ with respect to w_j at $\mathbf{0}$, leads to

$$d_j \simeq \frac{R(\Delta \boldsymbol{e}_j) - R(-\Delta \boldsymbol{e}_j)}{2\Delta}\,, \tag{S-1.35}$$

for a sufficiently small differential step size $\Delta > 0$, where $\boldsymbol{e}_j$ denotes a J-dimensional vector with its jth element being equal to one and the remaining elements being zero. By applying the previous equation twice, we obtain the following finite-difference approximation for the second-order partial derivative $d_{jj'}$ of $R(\boldsymbol{w})$ with respect to w_j and $w_{j'}$ at $\mathbf{0}$:

$$d_{jj'} \simeq \frac{R(\Delta \boldsymbol{e}_j + \Delta \boldsymbol{e}_{j'}) - R(-\Delta \boldsymbol{e}_j + \Delta \boldsymbol{e}_{j'}) - R(\Delta \boldsymbol{e}_j - \Delta \boldsymbol{e}_{j'}) + R(-\Delta \boldsymbol{e}_j - \Delta \boldsymbol{e}_{j'})}{4\Delta^2}\,. \tag{S-1.36}$$

To compute these approximations, we need $2J(J+1)+1$ system integrations, which is quadratic in terms of the number J of the underlying biochemical factors.

3.2 FD-HDMR

3.2.1 Polynomial approximation

The approximation of the SESI's and JESI's by means of (S-1.20) requires knowledge of the values of the α parameters associated with the polynomial approximation of the basis functions r, given by (S-1.19). This can be done by polynomial regression [11], as we explain next.

Our problem here is to estimate the parameters α, so that

$$r_j(w_j) = \widehat{r}_j(w_j) + \epsilon_j = \alpha_{j,1} w_j + \alpha_{j,2} w_j^2 + \epsilon_j,$$

and

$$\begin{aligned} r_{jj'}(w_j, w_{j'}) &= \widehat{r}_{jj'}(w_j, w_{j'}) + \epsilon_{jj'} \\ &= \alpha_{jj',1} w_j w_{j'} + \alpha_{jj',2} w_j^2 w_{j'} + \alpha_{jj',3} w_j w_{j'}^2 + \alpha_{jj',4} w_j^2 w_{j'}^2 + \epsilon_{jj'}, \end{aligned}$$

for every j, j', where the ϵ's are zero-mean random variables that model the errors of approximating the basis functions r by $\widehat{r}$. We can now use (S-1.3) to evaluate the basis functions r at a set $\{w_j(q), q \in S, j \in J\}$ of prespecified factor values around zero. Then, the least-squares error estimates $\widehat{\alpha}_{j,1}, \widehat{\alpha}_{j,2}$ of the parameters $\alpha_{j,1}, \alpha_{j,2}$ associated with the basis function $r_j(w_j)$ are given by [11]:

$$\widehat{\boldsymbol{\alpha}}_j = (\mathbb{W}_j^T\, \mathbb{W}_j)^{-1} \mathbb{W}_j^T\, \boldsymbol{r}_j\,,$$

where

$$\widehat{\boldsymbol{\alpha}}_j := \begin{bmatrix} \widehat{\alpha}_{j,1} \\ \widehat{\alpha}_{j,2} \end{bmatrix}_{2\times 1} \qquad \boldsymbol{r}_j := \begin{bmatrix} r_j(w_j(1)) \\ r_j(w_j(2)) \\ \vdots \\ r_j(w_j(S)) \end{bmatrix}_{S\times 1} \qquad \mathbb{W}_j := \begin{bmatrix} w_j(1) & w_j^2(1) \\ w_j(2) & w_j^2(2) \\ \vdots & \vdots \\ w_j(S) & w_j^2(S) \end{bmatrix}_{S\times 2},$$

provided that the matrix $\mathbb{W}_j^T \mathbb{W}_j$ is invertible (which is always true if no column of the $\mathbb{W}_j$ matrix is a linear combination of the other columns). On the other hand, the least-squares error estimates $\widehat{\alpha}_{jj',1}$, $\widehat{\alpha}_{jj',2}$, $\widehat{\alpha}_{jj',3}$, $\widehat{\alpha}_{jj',4}$ of the parameters $\alpha_{jj',1}$, $\alpha_{jj',2}$, $\alpha_{jj',3}$, $\alpha_{jj',4}$ associated with the basis function $\widehat{r}_{jj'}(w_j, w_{j'})$ are given by [11]:

$$\widehat{\boldsymbol{\alpha}}_{jj'} = (\mathbb{W}_{jj'}^T \mathbb{W}_{jj'})^{-1} \mathbb{W}_{jj'}^T \boldsymbol{r}_{jj'} ,$$

where

$$\widehat{\boldsymbol{\alpha}}_{jj'} := \begin{bmatrix} \widehat{\alpha}_{jj',1} \\ \widehat{\alpha}_{jj',2} \\ \widehat{\alpha}_{jj',3} \\ \widehat{\alpha}_{jj',4} \end{bmatrix}_{4\times 1} \qquad \boldsymbol{r}_{jj'} := \begin{bmatrix} r_{jj'}(w_j(1), w_{j'}(1)) \\ \vdots \\ r_{jj'}(w_j(1), w_{j'}(S)) \\ r_{jj'}(w_j(2), w_{j'}(1)) \\ \vdots \\ r_{jj'}(w_j(2), w_{j'}(S)) \\ \vdots \\ r_{jj'}(w_j(S), w_{j'}(1)) \\ \vdots \\ r_{jj'}(w_j(S), w_{j'}(S)) \end{bmatrix}_{S^2\times 1}$$

$$\mathbb{W}_{jj'} := \begin{bmatrix} w_j(1)w_{j'}(1) & w_j^2(1)w_{j'}(1) & w_j(1)w_{j'}^2(1) & w_j^2(1)w_{j'}^2(1) \\ \vdots & \vdots & \vdots & \vdots \\ w_j(1)w_{j'}(S) & w_j^2(1)w_{j'}(S) & w_j(1)w_{j'}^2(S) & w_j^2(1)w_{j'}^2(S) \\ w_j(2)w_{j'}(1) & w_j^2(2)w_{j'}(1) & w_j(2)w_{j'}^2(1) & w_j^2(2)w_{j'}^2(1) \\ \vdots & \vdots & \vdots & \vdots \\ w_j(2)w_{j'}(S) & w_j^2(2)w_{j'}(S) & w_j(2)w_{j'}^2(S) & w_j^2(2)w_{j'}^2(S) \\ w_j(S)w_{j'}(1) & w_j^2(S)w_{j'}(1) & w_j(S)w_{j'}^2(1) & w_j^2(S)w_{j'}^2(1) \\ \vdots & \vdots & \vdots & \vdots \\ w_j(S)w_{j'}(S) & w_j^2(S)w_{j'}(S) & w_j(S)w_{j'}^2(S) & w_j^2(S)w_{j'}^2(S) \end{bmatrix}_{S^2\times 4},$$

provided that the matrix $\mathbb{W}_{jj'}^T \mathbb{W}_{jj'}$ is invertible. Note that calculation of $\widehat{\boldsymbol{\alpha}}$ requires $J(J-1)S^2/2 + JS + 1$ system integrations, which is quadratic both in terms of the number J of biochemical factors and the number S of the samples per factor used in the regression.

3.2.2 Gauss-Hermite Integration

It is clear from (S-1.24) and (S-1.25) that evaluation of the SESI's and JESI's by (S-1.27) requires calculation of the expectations $\mathrm{E}[\psi_m(W_m)]$, $\mathrm{E}[\psi_{jm'}(w_j, W_m)]$, $\mathrm{E}[\psi_{mj}(W_m, w_j)]$, $\mathrm{E}[\psi_{mm'}(W_m, W_{m'})]$, $\mathrm{E}[e_j^2(w_j)]$, and $\mathrm{E}[e_{jj'}^2(w_j, w_{j'})]$ with respect to Gaussian distributions. We can evaluate these expectations by using Gauss-Hermite integration [12], as we explain next.

Let us consider the one-dimensional expectation:

$$E_1 = \mathrm{E}[\psi_1(W_1)] = \frac{1}{\lambda\sqrt{2\pi}} \int_{-\infty}^{\infty} \psi_1(w_1) e^{-w_1^2/2\lambda^2} dw_1.$$

If we set $w_1 = \sqrt{2}\lambda u_1$, then

$$E_1 = \frac{1}{\sqrt{\pi}} \int_{-\infty}^{\infty} \psi_1(\sqrt{2}\lambda u_1) e^{-u_1^2} du_1.$$

In this form, we can use the Gauss-Hermite integration procedure to approximate E_1 by

$$\widehat{E}_1 = \frac{1}{\sqrt{\pi}} \sum_{q=1}^{Q} \omega_q \psi_1(\sqrt{2}\lambda a_q),$$

where Q is the order of the approximation and a_q, ω_q are appropriately chosen abscissas and weights, respectively [12].

Likewise, by setting $w_1 = \sqrt{2}\lambda_1 u_1$ and $w_2 = \sqrt{2}\lambda_2 u_2$, we can write the two-dimensional expectation

$$E_2 = \mathrm{E}[\psi_2(W_1, W_2)] = \frac{1}{2\pi\lambda_1\lambda_2} \int_{-\infty}^{\infty} \int_{-\infty}^{\infty} \psi_2(w_1, w_2) e^{-w_1^2/2\lambda_1^2} e^{-w_2^2/2\lambda_2^2} dw_1 dw_2$$

in the form

$$E_2 = \frac{1}{\pi} \int_{-\infty}^{\infty} \int_{-\infty}^{\infty} \psi_2(\sqrt{2}\lambda_1 u_1, \sqrt{2}\lambda_2 u_2) e^{-u_1^2} e^{-u_2^2} du_1 du_2.$$

A two-step (first for u_1 and then for u_2) application of one-dimensional Gauss-Hermite integration results in the following approximation of E_2:

$$\widehat{E}_2 = \frac{1}{\pi} \sum_{q_1=1}^{Q} \sum_{q_2=1}^{Q} \omega_{q_1} \omega_{q_2} \psi_2(\sqrt{2}\lambda_1 a_{q_1}, \sqrt{2}\lambda_2 a_{q_2}).$$

It turns out that calculation of the expectations required by (S-1.27) involves $J(J-1)Q^2/2+ JQ+1$ system integrations, when Q is even, or $J(J-1)(Q-1)^2/2+J(Q-1)+1$ system integrations, when Q is odd, which is quadratic both in terms of the number J of biochemical factors and the number Q of points used by Gauss-Hermite integration.

3.3 ANOVA-HDMR

Approximating the sensitivity indices σ_j and η_j by (S-1.34) requires evaluation of the parameters α so that the functions $\widehat{\rho}$, given by (S-1.33), result in a sufficiently good approximation of the response function R by $\widehat{R}$, given by (S-1.31). Our problem here is to estimate the parameters α, so that

$$\rho_j(w_j) = \widehat{\rho}_j(w_j) + \epsilon_j = \alpha_{j,1}\frac{w_j}{\lambda_j} + \frac{\alpha_{j,2}}{\sqrt{2}}\left(\frac{w_j^2}{\lambda_j^2} - 1\right) + \epsilon_j, \tag{S-1.37}$$

and

$$\begin{aligned}\rho_{jj'}(w_j, w_{j'}) &= \widehat{\rho}_{jj'}(w_j, w_{j'}) + \epsilon_{jj'} \\ &= \alpha_{jj',1}\frac{w_j w_{j'}}{\lambda_j\lambda_{j'}} + \frac{\alpha_{jj',2}}{\sqrt{2}}\left(\frac{w_j^2}{\lambda_j^2} - 1\right)\frac{w_{j'}}{\lambda_{j'}} + \frac{\alpha_{jj',3}}{\sqrt{2}}\frac{w_j}{\lambda_j}\left(\frac{w_{j'}^2}{\lambda_{j'}^2} - 1\right) \\ &\quad + \frac{\alpha_{jj',4}}{2}\left(\frac{w_j^2}{\lambda_j^2} - 1\right)\left(\frac{w_{j'}^2}{\lambda_{j'}^2} - 1\right) + \epsilon_{jj'}\,,\end{aligned} \tag{S-1.38}$$

for every j, j', where the ϵ's are zero-mean random variables that model the errors in approximating the basis functions ρ by $\widehat{\rho}$. From (S-1.37), note that

$$\begin{aligned}\alpha_{j,1} &= \int_{-\infty}^{\infty}\frac{w_j}{\lambda_j}\rho_j(w_j)G_j(w_j)dw_j \\ \alpha_{j,2} &= \frac{1}{\sqrt{2}}\int_{-\infty}^{\infty}\left(\frac{w_j^2}{\lambda_j^2} - 1\right)\rho_j(w_j)G_j(w_j)dw_j\,,\end{aligned} \tag{S-1.39}$$

where $G_j(w_j)$ is the Gaussian probability density function

$$G_j(w_j) = \frac{1}{\sqrt{2\pi}\lambda_j}\,e^{-w_j^2/2\lambda_j^2}.$$

This is a consequence of the zero-mean Gaussianity of the biochemical factors and the orthonormality of the Hermite polynomials over the Gaussian distribution. Likewise, and from (S-1.38), we have that

$$\begin{aligned}
\alpha_{jj',1} &= \int_{-\infty}^{\infty} \frac{w_j}{\lambda_j}\frac{w_{j'}}{\lambda_{j'}}\,\rho_{jj'}(w_j, w_{j'})G_j(w_j)G_{j'}(w_{j'})dw_j dw_{j'} \\
\alpha_{jj',2} &= \frac{1}{\sqrt{2}}\int_{-\infty}^{\infty} \left(\frac{w_j^2}{\lambda_j^2} - 1\right)\frac{w_{j'}}{\lambda_{j'}}\,\rho_{jj'}(w_j, w_{j'})G_j(w_j)G_{j'}(w_{j'})dw_j dw_{j'} \\
\alpha_{jj',3} &= \frac{1}{\sqrt{2}}\int_{-\infty}^{\infty} \frac{w_j}{\lambda_j}\left(\frac{w_{j'}^2}{\lambda_{j'}^2} - 1\right)\rho_{jj'}(w_j, w_{j'})G_j(w_j)G_{j'}(w_{j'})dw_j dw_{j'} \\
\alpha_{jj',4} &= \frac{1}{2}\int_{-\infty}^{\infty} \left(\frac{w_j^2}{\lambda_j^2} - 1\right)\left(\frac{w_{j'}^2}{\lambda_{j'}^2} - 1\right)\rho_{jj'}(w_j, w_{j'})G_j(w_j)G_{j'}(w_{j'})dw_j dw_{j'}.
\end{aligned} \tag{S-1.40}$$

Finally,

$$\begin{aligned}
\alpha_{j,1} &= \mathrm{E}\left[\frac{W_j}{\lambda_j}R(\boldsymbol{W})\right] \\
\alpha_{j,2} &= \frac{1}{\sqrt{2}}\,\mathrm{E}\left[\left(\frac{W_j^2}{\lambda_j^2} - 1\right)R(\boldsymbol{W})\right] \\
\alpha_{jj',1} &= \mathrm{E}\left[\frac{W_j}{\lambda_j}\frac{W_{j'}}{\lambda_{j'}}R(\boldsymbol{W})\right] \\
\alpha_{jj',2} &= \frac{1}{\sqrt{2}}\,\mathrm{E}\left[\left(\frac{W_j^2}{\lambda_j^2} - 1\right)\frac{W_{j'}}{\lambda_{j'}}R(\boldsymbol{W})\right] \\
\alpha_{jj',3} &= \frac{1}{\sqrt{2}}\,\mathrm{E}\left[\frac{W_j}{\lambda_j}\left(\frac{W_{j'}^2}{\lambda_{j'}^2} - 1\right)R(\boldsymbol{W})\right] \\
\alpha_{jj',4} &= \frac{1}{2}\,\mathrm{E}\left[\left(\frac{W_j^2}{\lambda_j^2} - 1\right)\left(\frac{W_{j'}^2}{\lambda_{j'}^2} - 1\right)R(\boldsymbol{W})\right],
\end{aligned} \tag{S-1.41}$$

by virtue of (S-1.10), (S-1.39), and (S-1.40).

As a consequence of the previous analysis, to determine the parameters α, we need to evaluate the expectations in (S-1.41). We can do this by Monte Carlo estimation based on a Latin hypercube sampling strategy, which leads to a more efficient implementation than standard Monte Carlo sampling [13, 14]. In particular, we can generate L Latin hypercube Gaussian samples $\boldsymbol{w}^{(l)} = \{w_1^{(l)}, w_2^{(l)}, \ldots, w_J^{(l)}\}$,

$l = 1, 2, \ldots, L$, evaluate the responses $R(\boldsymbol{w}^{(l)})$, for $l = 1, 2, \ldots, L$, and set

$$\alpha_{j,1} \simeq \widehat{\alpha}_{j,1} := \frac{1}{L} \sum_{l=1}^{L} \frac{w_j^{(l)}}{\lambda_j} R(\boldsymbol{w}^{(l)})$$

$$\alpha_{j,2} \simeq \widehat{\alpha}_{j,2} := \frac{1}{\sqrt{2}} \frac{1}{L} \sum_{l=1}^{L} \left(\frac{[w_j^{(l)}]^2}{\lambda_j^2} - 1 \right) R(\boldsymbol{w}^{(l)})$$

$$\alpha_{jj',1} \simeq \widehat{\alpha}_{jj',1} := \frac{1}{L} \sum_{l=1}^{L} \frac{w_j^{(l)}}{\lambda_j} \frac{w_{j'}^{(l)}}{\lambda_{j'}} R(\boldsymbol{w}^{(l)})$$

$$\alpha_{jj',2} \simeq \widehat{\alpha}_{jj',2} := \frac{1}{\sqrt{2}} \frac{1}{L} \sum_{l=1}^{L} \left(\frac{[w_j^{(l)}]^2}{\lambda_j^2} - 1 \right) \frac{w_{j'}^{(l)}}{\lambda_{j'}} R(\boldsymbol{w}^{(l)})$$

$$\alpha_{jj',3} \simeq \widehat{\alpha}_{jj',3} := \frac{1}{\sqrt{2}} \frac{1}{L} \sum_{l=1}^{L} \frac{w_j^{(l)}}{\lambda_j} \left(\frac{[w_{j'}^{(l)}]^2}{\lambda_{j'}^2} - 1 \right) R(\boldsymbol{w}^{(l)})$$

$$\alpha_{jj',4} \simeq \widehat{\alpha}_{jj',4} := \frac{1}{2} \frac{1}{L} \sum_{l=1}^{L} \left(\frac{[w_j^{(l)}]^2}{\lambda_j^2} - 1 \right) \left(\frac{[w_{j'}^{(l)}]^2}{\lambda_{j'}^2} - 1 \right) R(\boldsymbol{w}^{(l)}). \tag{S-1.42}$$

Clearly, implementation of (S-1.42) requires L system integrations.

The problem with Monte Carlo estimation is that, most often, it requires a large number of system integrations to produce sufficiently accurate estimates for the α parameters. As a consequence, it is a computationally inefficient method for estimating α. An alternative approach is to use the previous L samples $\{\boldsymbol{w}^{(l)}, l = 1, 2, \ldots, L\}$ and estimate the α parameters by polynomial regression, as we did in Section 3.2.1. We discuss this approach in the following.

As a consequence of (S-1.31) and (S-1.33), the polynomial regression problem amounts to estimating $\widehat{\rho}_0$ and the parameters α, so that

$$\begin{aligned} R(\boldsymbol{w}) &= \widehat{R}(\boldsymbol{w}) + \epsilon \\ &= \widehat{\rho}_0 + \sum_{j=1}^{J} \alpha_{j,1} \frac{w_j}{\lambda_j} + \frac{\alpha_{j,2}}{\sqrt{2}} \left(\frac{w_j^2}{\lambda_j^2} - 1 \right) \\ &\quad + \sum_{j=1}^{J-1} \sum_{j'=j+1}^{J} \alpha_{jj',1} \frac{w_j w_{j'}}{\lambda_j \lambda_{j'}} + \frac{\alpha_{jj',2}}{\sqrt{2}} \left(\frac{w_j^2}{\lambda_j^2} - 1 \right) \frac{w_{j'}}{\lambda_{j'}} \end{aligned}$$

$$+\sum_{j=1}^{J-1}\sum_{j'=j+1}^{J}\frac{\alpha_{jj',3}}{\sqrt{2}}\frac{w_j}{\lambda_j}\left(\frac{w_{j'}^2}{\lambda_{j'}^2}-1\right)+\frac{\alpha_{jj',4}}{2}\left(\frac{w_j^2}{\lambda_j^2}-1\right)\left(\frac{w_{j'}^2}{\lambda_{j'}^2}-1\right)+\epsilon\,,$$

where ϵ is a zero-mean random variable that models the errors of approximating the response function R by $\widehat{R}$. In this case, the least-squares error estimate $\widehat{\boldsymbol{\alpha}}$ of the parameters α are given by

$$\widehat{\boldsymbol{\alpha}}=(\mathbb{W}^T\mathbb{W})^{-1}\mathbb{W}^T\boldsymbol{\rho}\,, \tag{S-1.43}$$

where

$$\widehat{\boldsymbol{\alpha}}:=\begin{bmatrix}\widehat{\rho}_0\\ \widehat{\alpha}_{1,1}\\ \vdots\\ \widehat{\alpha}_{1,2}\\ \vdots\\ \widehat{\alpha}_{(J-1)J,4}\end{bmatrix}_{(2J^2+1)\times 1}\qquad \boldsymbol{\rho}:=\begin{bmatrix}R(\boldsymbol{w}^{(1)})\\ R(\boldsymbol{w}^{(2)})\\ \vdots\\ R(\boldsymbol{w}^{(L)})\end{bmatrix}_{L\times 1}$$

$$\mathbb{W}:=\begin{bmatrix}1 & w_1^{(1)}/\lambda_1 & \cdots & [(w_1^{(1)}/\lambda_1)^2-1]/\sqrt{2} & \cdots & [(w_{J-1}^{(1)}/\lambda_{J-1})^2-1][(w_J^{(1)}/\lambda_J)^2-1]/2\\ 1 & w_1^{(2)}/\lambda_1 & \cdots & [(w_1^{(2)}/\lambda_1)^2-1]/\sqrt{2} & \cdots & [(w_{J-1}^{(2)}/\lambda_{J-1})^2-1][(w_J^{(2)}/\lambda_J)^2-1]/2\\ \vdots & \vdots & \vdots & \vdots & \vdots & \vdots\\ 1 & w_1^{(L)}/\lambda_1 & \cdots & [(w_1^{(L)}/\lambda_1)^2-1]/\sqrt{2} & \cdots & [(w_{J-1}^{(L)}/\lambda_{J-1})^2-1][(w_J^{(L)}/\lambda_J)^2-1]/2\end{bmatrix}_{L\times(2J^2+1)}, \tag{S-1.44}$$

provided that the matrix $\mathbb{W}^T\mathbb{W}$ is invertible. Note that calculation of $\widehat{\boldsymbol{\alpha}}$ requires the same number L of system integrations as Monte Carlo estimation by (S-1.42).

It is not difficult to see from (S-1.43) that, if $\widehat{\boldsymbol{\alpha}}_{\text{MC}}$ is the Monte Carlo estimate of $\widehat{\rho}_0$ and of the parameters α, given by [recall (S-1.10)]

$$\widehat{\rho}_0=\rho_0=\mathrm{E}[R(\boldsymbol{W})]\simeq\frac{1}{L}\sum_{l=1}^{L}R(\boldsymbol{w}^{(l)}),$$

and (S-1.42), respectively, then

$$\widehat{\boldsymbol{\alpha}}_{\text{MC}}=\frac{1}{L}\,\mathbb{W}^T\boldsymbol{\rho}=\frac{1}{L}\,\mathbb{W}^T\mathbb{W}\,\widehat{\boldsymbol{\alpha}}\,.$$

Moreover,

$$\lim_{L\to\infty}\frac{1}{L}\mathbb{W}^T\mathbb{W}=\mathbb{I}, \tag{S-1.45}$$

where $\mathbb{I}$ is the identity matrix, by virtue of the biorthonormality conditions given by (S-1.32) and the fact that the Monte Carlo estimate $\sum_{l=1}^{L}f(x^{(l)})/L$ converges to the integral $\int_{-\infty}^{\infty}f(x)\pi(x)dx$, as $L\to\infty$, provided that $x^{(l)}$, $l=1,2,\ldots,L$, are samples independently drawn from the probability density function

$\pi(x)$. As a consequence, the Monte Carlo estimate $\widehat{\boldsymbol{\alpha}}_{\text{MC}}$ and the regression estimate $\widehat{\boldsymbol{\alpha}}$ are identical in the limit as the number of Monte Carlo samples grows to infinity. Since $\boldsymbol{\alpha}$ is obtained by minimizing the least-squares error between R and $\widehat{R}$, we expect that the regression estimate $\boldsymbol{\alpha}$ of the parameters α will be more preferable than the Monte Carlo estimate $\widehat{\boldsymbol{\alpha}}_{\text{MC}}$, in the sense that, for a relatively small number of Monte Carlo samples, $\boldsymbol{\alpha}$ may produce a better fit $\widehat{R}$ of the response function R than the one produced by $\widehat{\boldsymbol{\alpha}}_{\text{MC}}$. Finally, note from (S-1.45) that, for a sufficiently large number of Monte Carlo samples, $\mathbb{W}^T\mathbb{W}$ is approximately equal to the identity matrix multiplied by L, which effectively reduces the risk of singularity when evaluating the inverse matrix $(\mathbb{W}^T\mathbb{W})^{-1}$ in (S-1.43). Therefore, if $\mathbb{W}^T\mathbb{W}$ turns out to be singular for a chosen value of L, the user needs to increase L until a nonsingular matrix $\mathbb{W}^T\mathbb{W}$ is obtained.

References

1. Rabitz H, Aliş ÖF, Shorter J, Shim K: **Efficient input-output model representations**. *Comput. Phys. Commun.* 1999, **117**:11–20.
2. Rabitz H, Aliş ÖF: **General foundations of high-dimensional model representations**. *J. Math. Chem.* 1999, **25**:197–233.
3. Li G, Rosenthal C, Rabitz H: **High dimensional model representations**. *J. Phys. Chem. A* 2001, **105**:7765–7777.
4. Sobol' IM: **Theorems and examples on high-dimensional model representation**. *Reliab. Eng. Syst. Safe.* 2003, **79**:187–193.
5. Sobol' IM: **Global sensitivity indices for nonlinear mathematical models and their Monte Carlo estimates**. *Math. Comput. Simulat.* 2001, **55**:271–280.
6. Chen W, Jin R, Sudjianto A: **Analytical variance-based global sensitivity analysis in simulation-based design under uncertainty**. *J. Mech. Design* 2005, **127**:875–886.
7. Archer GEB, Saltelli A, Sobol IM: **Sensitivity measures, ANOVA-like techniques and the use of bootstrap**. *J. Statist. Comput. Simul.* 1997, **58**:99–120.
8. Owen AB: **Latin supercube sampling for very high-dimensional simulations**. *ACM T. Model. Comput. S.* 1998, **8**:71–102.
9. Cacuci DG: *Sensitivity and Uncertainty Analysis*, *Volume I. Theory*. Boca Raton: Chapman & Hall/CRC 2003.
10. Varma A, Morbidelli M, Wu H: *Parametric Sensitivity in Chemical Systems*. Cambridge, UK: Cambridge University Press 1999.
11. Montgomery DC, Peck EA, Vining GG: *Introduction to Linear Regression Analysis*. New York: John Wiley, 3rd edition 2001.
12. Press WH, Teukolsky SA, Vetterling WT, Flannery BP: *Numerical Recipes: The Art of Scientific Computing*. New York: Cambridge University Press, 3rd edition 2007.
13. Stein M: **Large sample properties of simulations using Latin hypercube sampling**. *Technometrics* 1987, **29**:143–151.
14. McKay MD, Conover WJ, Beckman RJ: **A Comparison of three methods for selecting values of input variables in the analysis of output from a computer code**. *Technometrics* 2000, **42**:55–61.
