# Supplementary material for: A comparison of approximation techniques for variance-based sensitivity analysis of biochemical reaction systems
Source: BMC Bioinformatics. 2010 May 12;11:246. doi: 10.1186/1471-2105-11-246 (PMC2894038; doi:10.1186/1471-2105-11-246)
Supplement: Additional file 2 — MAPK signaling cascade model. This file lists the biochemical reactions associated with the MAPK signaling cascade model and provides nominal values for the normalized reaction rate constants and initial molecular concentrations. [file 1471-2105-11-246-S2.PDF]

ADDITIONAL FILE 2

A comparison of approximation techniques for variance-based sensitivity analysis of biochemical reaction systems

MAPK SIGNALING CASCADE MODEL

Hong-Xuan Zhang[1] **and John Goutsias***[1]

[1] Whitaker Biomedical Engineering Institute, The Johns Hopkins University, Baltimore, MD 21218, USA

* Corresponding author

Email: HXZ: hxzhang@jhu.edu, JG: goutsias@jhu.edu

In this document, we list the biochemical reactions associated with the MAPK signaling cascade model we consider in the Main text and provide nominal values for the normalized reaction rate constants (measured in s^{-1}) and the initial molecular concentrations (measured in molecules/cell). We depict this model in Figure 1 of the Main text. We have adopted the data from Schoeberl *et al.* [1], with a few rate constant values updated from the "JWS Online Cellular Systems Modeling" web site (http://jjj.biochem.sun.ac.za). The first reaction in the model depicted in Figure 1 of the Main text compensates for Ras-GTP synthesis which, in reality, is accomplished by a complex epidermal growth factor (EGF)-induced signalling pathway [1]. We have set the reaction rate constant of Ras-GTP synthesis equal to $3s^{-1}$. This value results in an ERK-PP concentration profile that is similar to the one reported by Schoeberl *et al.* [1], with 50ng/ml EGF.

Reactions

No.	Reaction	Rate Constant (s^{-1})
**1**	$\emptyset \rightarrow$ Ras-GTP	$\kappa_1 = 3$
	Ras-GTP $\rightarrow \emptyset$	$\kappa_2 = 0$
**2**	Ras-GTP + Raf $\rightarrow$ Raf-Ras-GTP	$\kappa_3 = 1.6605 \times 10^{-6}$
	Raf-Ras-GTP $\rightarrow$ Raf + Ras-GTP	$\kappa_4 = 5.3 \times 10^{-3}$
**3**	Raf-Ras-GTP $\rightarrow$ Raf* + Ras-GTP*	$\kappa_5 = 1$
	Raf* + Ras-GTP* $\rightarrow$ Raf-Ras-GTP	$\kappa_6 = 1.1624 \times 10^{-6}$
**4**	Raf* + Pho1 $\rightarrow$ Raf*-Pho1	$\kappa_7 = 1.1790 \times 10^{-4}$
	Raf*-Pho1 $\rightarrow$ Raf* + Pho1	$\kappa_8 = 0.2$
**5**	Raf*-Pho1 $\rightarrow$ Raf + Pho1	$\kappa_9 = 1$
	Raf + Pho1 $\rightarrow$ Raf*-Pho1	$\kappa_{10} = 0$
**6**	MEK + Raf* $\rightarrow$ MEK-Raf*	$\kappa_{11} = 1.9428 \times 10^{-5}$
	MEK-Raf* $\rightarrow$ MEK + Raf*	$\kappa_{12} = 3.3 \times 10^{-2}$
**7**	MEK-Raf* $\rightarrow$ MEK-P + Raf*	$\kappa_{13} = 3.5$
	MEK-P + Raf* $\rightarrow$ MEK-Raf*	$\kappa_{14} = 0$
**8**	MEK-P + Raf* $\rightarrow$ MEK-P-Raf*	$\kappa_{15} = 1.9428 \times 10^{-5}$
	MEK-P-Raf* $\rightarrow$ MEK-P + Raf*	$\kappa_{16} = 3.3 \times 10^{-2}$
**9**	MEK-P-Raf* $\rightarrow$ MEK-PP + Raf*	$\kappa_{17} = 2.9$
	MEK-PP + Raf* $\rightarrow$ MEK-P-Raf*	$\kappa_{18} = 0$
**10**	MEK-PP + Pho2 $\rightarrow$ MEK-PP-Pho2	$\kappa_{19} = 2.3746 \times 10^{-5}$
	MEK-PP-Pho2 $\rightarrow$ MEK-PP + Pho2	$\kappa_{20} = 0.8$
**11**	MEK-PP-Pho2 $\rightarrow$ MEK-P + Pho2	$\kappa_{21} = 5.8 \times 10^{-2}$
	MEK-P + Pho2 $\rightarrow$ MEK-PP-Pho2	$\kappa_{22} = 0$
**12**	MEK-P + Pho2 $\rightarrow$ MEK-P-Pho2	$\kappa_{23} = 4.4835 \times 10^{-7}$
	MEK-P-Pho2 $\rightarrow$ MEK-P + Pho2	$\kappa_{24} = 0.5$
**13**	MEK-P-Pho2 $\rightarrow$ MEK + Pho2	$\kappa_{25} = 5.8 \times 10^{-2}$
	MEK + Pho2 $\rightarrow$ MEK-P-Pho2	$\kappa_{26} = 0$
**14**	ERK + MEK-PP $\rightarrow$ ERK-MEK-PP	$\kappa_{27} = 8.8673 \times 10^{-5}$
	ERK-MEK-PP $\rightarrow$ ERK + MEK-PP	$\kappa_{28} = 1.833 \times 10^{-2}$
**15**	ERK-MEK-PP $\rightarrow$ ERK-P + MEK-PP	$\kappa_{29} = 16$
	ERK-P + MEK-PP $\rightarrow$ ERK-MEK-PP	$\kappa_{30} = 0$
**16**	ERK-P + MEK-PP $\rightarrow$ ERK-P-MEK-PP	$\kappa_{31} = 8.8673 \times 10^{-5}$
	ERK-P-MEK-PP $\rightarrow$ ERK-P + MEK-PP	$\kappa_{32} = 1.833 \times 10^{-2}$
**17**	ERK-P-MEK-PP $\rightarrow$ ERK-PP + MEK-PP	$\kappa_{33} = 5.7$
	ERK-PP + MEK-PP $\rightarrow$ ERK-P-MEK-PP	$\kappa_{34} = 0$
**18**	ERK-PP + Pho3 $\rightarrow$ ERK-PP-Pho3	$\kappa_{35} = 2.3414 \times 10^{-5}$
	ERK-PP-Pho3 $\rightarrow$ ERK-PP + Pho3	$\kappa_{36} = 0.6$
**19**	ERK-PP-Pho3 $\rightarrow$ ERK-P + Pho3	$\kappa_{37} = 0.246$
	ERK-P + Pho3 $\rightarrow$ ERK-PP-Pho3	$\kappa_{38} = 0$
**20**	ERK-P + Pho3 $\rightarrow$ ERK-P-Pho3	$\kappa_{39} = 8.3027 \times 10^{-6}$
	ERK-P-Pho3 $\rightarrow$ ERK-P + Pho3	$\kappa_{40} = 0.5$
**21**	ERK + Pho3 $\rightarrow$ ERK-P-Pho3	$\kappa_{41} = 0$
	ERK-P-Pho3 $\rightarrow$ ERK + Pho3	$\kappa_{42} = 0.246$

Initial Concentrations

No.	species	molecules/cell
**1**	Ras-GTP	7.20×10^4
**2**	Raf	4.00×10^4
**3**	Raf-Ras-GTP	0
**4**	Raf*	0
**5**	Pho1	4.00×10^4
**6**	Raf*-Pho1	0
**7**	MEK	2.10×10^8
**8**	MEK-Raf*	0
**9**	MEK-P	0
**10**	MEK-P-Raf*	0
**11**	MEK-PP	0
**12**	Pho2	4.00×10^4
**13**	MEK-PP-Pho2	0
**14**	MEK-P-Pho2	0
**15**	ERK	2.21×10^7
**16**	ERK-MEK-PP	0
**17**	ERK-P	0
**18**	ERK-P-MEK-PP	0
**19**	ERK-PP	0
**20**	Pho3	1.00×10^7
**21**	ERK-PP-Pho3	0
**22**	ERK-P-Pho3	0
**23**	Ras-GTP*	0

References

1. Schoeberl B, Eichler-Jonsson C, Gilles ED, Müller G: **Computational modeling of the dynamics of the MAP kinase cascade activated by surface and internalized EGF receptors**. *Nat. Biotechnol.* 2002, **20**:370–375.
