# Supplementary material for: A comparison of approximation techniques for variance-based sensitivity analysis of biochemical reaction systems
Source: BMC Bioinformatics. 2010 May 12;11:246. doi: 10.1186/1471-2105-11-246 (PMC2894038; doi:10.1186/1471-2105-11-246)
Supplement: Additional file 3 — SOSA-based sensitivity analysis results. This file summarizes the SOSA-based sensitivity analysis results for the three response characteristics (duration, integrated response, and strength) of ERK-PP activity in the MAPK signaling cascade obtained by the five approximation methods discussed in the paper. [file 1471-2105-11-246-S3.PDF]

ADDITIONAL FILE 3

A comparison of approximation techniques for variance-based sensitivity analysis of biochemical reaction systems

SOSA-BASED SENSITIVITY ANALYSIS RESULTS

Hong-Xuan Zhang[1] **and John Goutsias***[1]

[1] Whitaker Biomedical Engineering Institute, The Johns Hopkins University, Baltimore, MD 21218, USA

* Corresponding author

Email: HXZ: hxzhang@jhu.edu, JG: goutsias@jhu.edu

In this document, we provide the SOSA-based sensitivity analysis results for the three response characteristics (duration, integrated response, and strength) of ERK-PP activity in the MAPK signaling cascade obtained by the five techniques (MC, DA, PA, GHI, and OHA) considered in the Main text and for four fluctuation levels ($\lambda = 0.1, 0.2, 0.3, 0.4$) in the values of the standard chemical potentials associated with the molecular species. The results are given in percentages and have been truncated to the nearest integer. Only results that correspond to SESI or JESI values obtained by MC that are at least 5% are shown. Bold species numbers indicate SESI or JESI values that are at least 10%. According to our discussion in the Main text, these species are classified by the variance-based sensitivity analysis method to be *singularly influential* (if the SESI value is at least 10% but the JESI value is below 10%), *jointly influential* (if the JESI value is at least 10% but the SESI value is below 10%), and *singularly/jointly influential* (if both the SESI and JESI values are at least 10%). The remaining molecular species are deemed to be *noninfluential*.

Table S-3.1. SOSA-based sensitivity analysis results for the *duration* of ERK-PP activity.

SESI - DURATION ($\lambda = 0.1$)						JESI - DURATION ($\lambda = 0.1$)					
**Species**	**MC**	**DA**	**PA**	**GHI**	**OHA**	**Species**	**MC**	**DA**	**PA**	**GHI**	**OHA**
**5**	38	37	38	34	38	5	1	0	0	0	0
**7**	23	25	23	25	23	7	0	0	0	0	0
**14**	17	17	19	19	18	14	0	0	0	0	0
**SESI - DURATION ($\lambda = 0.2$)**						**JESI - DURATION ($\lambda = 0.2$)**					
**Species**	**MC**	**DA**	**PA**	**GHI**	**OHA**	**Species**	**MC**	**DA**	**PA**	**GHI**	**OHA**
**5**	36	35	36	37	37	5	4	1	1	1	1
**7**	20	24	22	20	22	7	2	1	1	1	1
**14**	15	16	17	19	16	14	1	0	0	0	0
**SESI - DURATION ($\lambda = 0.3$)**						**JESI - DURATION ($\lambda = 0.3$)**					
**Species**	**MC**	**DA**	**PA**	**GHI**	**OHA**	**Species**	**MC**	**DA**	**PA**	**GHI**	**OHA**
**5**	36	33	36	33	36	5	3	3	2	3	2
**7**	20	23	20	21	21	7	1	1	1	1	1
**14**	15	16	14	14	15	14	1	1	1	1	1
18	5	4	5	6	5	18	1	2	2	2	1
**SESI - DURATION ($\lambda = 0.4$)**						**JESI - DURATION ($\lambda = 0.4$)**					
**Species**	**MC**	**DA**	**PA**	**GHI**	**OHA**	**Species**	**MC**	**DA**	**PA**	**GHI**	**OHA**
**5**	34	31	27	32	33	5	5	4	4	5	5
**7**	19	21	20	18	19	7	3	2	2	2	3
12	5	4	5	4	6	12	1	1	0	0	1
**14**	15	15	13	11	15	14	1	1	1	1	1

Table S-3.2. SOSA-based sensitivity analysis results for the *integrated response* of ERK-PP activity.

**SESI - I-RESPONSE ($\lambda = 0.1$)**						**JESI - I-RESPONSE ($\lambda = 0.1$)**					
**Species**	**MC**	**DA**	**PA**	**GHI**	**OHA**	**Species**	**MC**	**DA**	**PA**	**GHI**	**OHA**
**5**	46	47	47	47	47	5	1	0	0	0	0
**7**	23	23	23	23	23	7	0	0	0	0	0
9	9	9	9	9	9	9	1	0	0	0	0
**14**	11	12	12	12	12	14	0	0	0	0	0
**SESI - I-RESPONSE ($\lambda = 0.2$)**						**JESI - I-RESPONSE ($\lambda = 0.2$)**					
**Species**	**MC**	**DA**	**PA**	**GHI**	**OHA**	**Species**	**MC**	**DA**	**PA**	**GHI**	**OHA**
**5**	47	46	50	49	46	5	7	1	2	5	5
**7**	19	23	21	20	21	7	4	0	1	2	2
9	8	9	9	8	9	9	3	0	1	2	3
14	8	12	9	9	9	14	1	0	0	0	0
**SESI - I-RESPONSE ($\lambda = 0.3$)**						**JESI - I-RESPONSE ($\lambda = 0.3$)**					
**Species**	**MC**	**DA**	**PA**	**GHI**	**OHA**	**Species**	**MC**	**DA**	**PA**	**GHI**	**OHA**
**5**	47	45	50	52	44	**5**	14	2	16	14	15
**7**	16	23	15	15	16	7	7	1	6	5	6
9	9	9	9	8	9	9	5	1	6	5	7
**SESI - I-RESPONSE ($\lambda = 0.4$)**						**JESI - I-RESPONSE ($\lambda = 0.4$)**					
**Species**	**MC**	**DA**	**PA**	**GHI**	**OHA**	**Species**	**MC**	**DA**	**PA**	**GHI**	**OHA**
**5**	45	44	45	48	46	**5**	16	3	22	17	15
**7**	15	22	13	14	15	7	8	1	8	7	7
9	9	8	9	10	9	9	7	1	8	7	7

Table S-3.3. SOSA-based sensitivity analysis results for the *strength* of ERK-PP activity.

**SESI - STRENGTH ($\lambda = 0.1$)**						**JESI - STRENGTH ($\lambda = 0.1$)**					
**Species**	**MC**	**DA**	**PA**	**GHI**	**OHA**	**Species**	**MC**	**DA**	**PA**	**GHI**	**OHA**
**5**	40	41	40	38	41	5	1	0	0	0	0
**7**	13	11	14	8	13	7	1	0	0	0	0
**9**	26	26	27	29	26	9	1	0	0	0	0
17	5	6	5	5	6	17	0	0	0	0	0
21	6	6	5	8	6	21	0	0	0	0	0
**SESI - STRENGTH ($\lambda = 0.2$)**						**JESI - STRENGTH ($\lambda = 0.2$)**					
**Species**	**MC**	**DA**	**PA**	**GHI**	**OHA**	**Species**	**MC**	**DA**	**PA**	**GHI**	**OHA**
**5**	40	38	47	46	35	**5**	18	2	10	18	17
**7**	10	10	11	11	10	7	9	1	4	6	7
**9**	15	24	17	16	17	9	9	1	4	6	9
**SESI - STRENGTH ($\lambda = 0.3$)**						**JESI - STRENGTH ($\lambda = 0.3$)**					
**Species**	**MC**	**DA**	**PA**	**GHI**	**OHA**	**Species**	**MC**	**DA**	**PA**	**GHI**	**OHA**
**5**	41	35	44	49	34	**5**	27	3	30	28	29
7	8	9	7	7	8	**7**	15	1	11	9	13
**9**	10	22	10	9	10	9	9	1	10	9	9
22	1	0	0	0	1	22	6	5	4	4	7
**SESI - STRENGTH ($\lambda = 0.4$)**						**JESI - STRENGTH ($\lambda = 0.4$)**					
**Species**	**MC**	**DA**	**PA**	**GHI**	**OHA**	**Species**	**MC**	**DA**	**PA**	**GHI**	**OHA**
**5**	40	31	40	41	39	**5**	26	5	35	29	26
7	8	8	7	8	8	**7**	13	2	12	11	13
9	9	20	8	10	9	**9**	11	2	11	10	11
22	2	0	1	1	2	22	6	8	5	5	7
